# High-throughput gas separation by flexible metal–organic frameworks with fast gating and thermal management capabilities

**DOI:** 10.1038/s41467-020-17625-3

**Published:** 2020-08-03

**Authors:** Shotaro Hiraide, Yuta Sakanaka, Hiroshi Kajiro, Shogo Kawaguchi, Minoru T. Miyahara, Hideki Tanaka

**Affiliations:** 1 Department of Chemical Engineering, Kyoto University, Nishikyo, Kyoto, 615-8510 Japan; 2 Nippon Steel Corporation, 20-1 Shintomi, Futtsu Chiba, 293-8511 Japan; 3 Japan Synchrotron Radiation Research Institute (JASRI), SPring-8, 1-1-1 Kouto, Sayo Hyogo, 679-5198 Japan; 4 Research Initiative for Supra-Materials (RISM), Shinshu University, 4-17-1 Wakasato, Nagano, 380-8553 Japan

**Keywords:** Chemical engineering, Metal-organic frameworks

## Abstract

Establishing new energy-saving systems for gas separation using porous materials is indispensable for ensuring a sustainable future. Herein, we show that ELM-11 ([Cu(BF_4_)_2_(4,4′-bipyridine)_2_]_n_), a member of flexible metal–organic frameworks (MOFs), exhibits rapid responsiveness to a gas feed and an ‘intrinsic thermal management’ capability originating from a structural deformation upon gas adsorption (gate-opening). These two characteristics are suitable for developing a pressure vacuum swing adsorption (PVSA) system with rapid operations. A combined experimental and theoretical study reveals that ELM-11 enables the high-throughput separation of CO_2_ from a CO_2_/CH_4_ gas mixture through adiabatic operations, which are extreme conditions in rapid pressure vacuum swing adsorption. We also propose an operational solution to the ‘slipping-off’ problem, which is that the flexible MOFs cannot adsorb target molecules when the partial pressure of the target gas decreases below the gate-opening pressure. Furthermore, the superiority of our proposed system over conventional systems is demonstrated.

## Abstract (web summary)

Separation processes in industry use substantial energy and energy-efficient purification systems should be developed for sustainability. Here the authors report a flexible metal–organic framework for high-throughput separation of CO_2_ from a CO_2_/CH_4_ gas mixture in a pressure vacuum swing adsorption system.

## Introduction

Half of the USA’s industrial energy is consumed in separation processes, among which 49% of separation costs are for distillation^1^. To ensure a sustainable future, it is necessary to establish new-energy-saving purification systems, such as gas permeation with membranes and gas adsorption with porous materials. Pressure vacuum swing adsorption (PVSA) and temperature swing adsorption (TSA) processes are much more energy efficient than distillation. However, it is difficult for these techniques to achieve high-throughput separation, which requires system enlargement, because they cause pressure loss and crushing of adsorbents at the bottom of the adsorption column. One potential solution to this problem is to rapidly operate a PVSA process to increase the flow rate of the gas without enlarging the system size. This technique, so-called rapid PVSA, is currently widely investigated and recognized as a possible strategy^2–4^. However, the rapid PVSA process is also limited in that the short-term cycle makes the system more adiabatic; thus, the generation of heat of adsorption and resulting amount of the decrease in adsorption is more serious than in the case of the normal PVSA process. The coexistence of adsorbents and phase change materials as latent heat storage mediums in a column may solve this problem^5^, though such a coexistence requires system enlargement. Therefore, to achieve a high-throughput separation system, innovative adsorption materials that exhibit a large loading capacity, high selectivity, and minimal heat of adsorption are required. Such ground-breaking materials must have completely different characteristics than conventional materials because high affinity and low heat of adsorption are essentially conflicting.

**Fig. 1:**
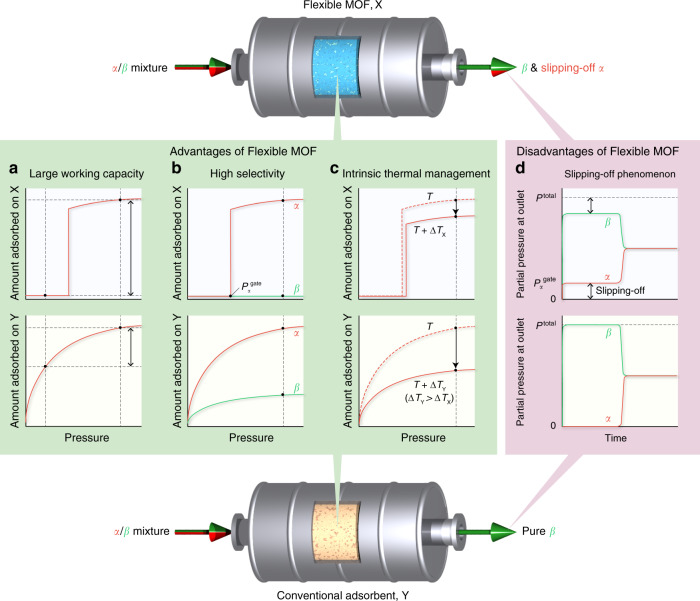
Advantages and disadvantages of flexible metal–organic frameworks (MOFs). **a** Stepwise change in the amount adsorbed due to the gate-opening of the flexible MOF, X, can provide a larger working capacity when the same pressure swing is considered for the flexible MOF and the conventional adsorbent, Y. **b** For a gas mixture adsorption (components *α* and *β*), the flexible MOF can give higher selectivity for component *α* than the conventional adsorbent can give, because the gate-opening is a kind of molecular recognition; in this case, the flexible MOF can only accommodate component *α* and shows gate-opening. **c** When an adiabatic gas adsorption is considered, the temperature rise of the system for the flexible MOF, Δ*T*_X_, is smaller than that for the conventional adsorbent, Δ*T*_Y_, and the resulting decrease in the adsorption amount can be suppressed because of the smaller net heat of adsorption of the flexible MOF owing to its intrinsic thermal management capability. **d** The flexible MOF cannot adsorb component *α* when the partial pressure of *α* in a gas mixture decreases below the inherent gate-opening pressure of the flexible MOF, *P*_*α*_^gate^. Thus, a pure effluent gas (component *β*) cannot be obtained at the end of the adsorption column (“slipping-off” phenomenon).

For a dozen years, metal–organic frameworks (MOFs) have been extensively studied as promising porous materials, as their designable framework structures can provide desirable adsorption properties. Moreover, flexible MOFs have attracted great attention as a result of their peculiar “gate-opening” and “breathing” behaviour, which is a structural transition phenomenon induced by guest adsorption^6–11^. The gate-opening and breathing effects induce stepwise change in the amount adsorbed at a specific gas pressure, which engenders larger working capacities and higher selectivities than the conventional adsorbents do. This is schematically illustrated in Fig. 1a,b; therein, the classical Langmuir-type adsorption isotherm is shown. Furthermore, it has been recently reported that flexible MOFs possess “intrinsic thermal management” capabilities^12,13^. Namely, the exothermic heat associated with guest adsorption is partially offset by the endothermic expansion of the host framework during gate-opening. The opposite phenomenon occurs during gate-closing based on the relationship between the endothermic heat of desorption of the guest and the exothermic shrinkage of the host. These effects are not observed for conventional adsorbents with rigid frameworks, but they are desired features for the rapid PVSA process: resulting from the suppressed heating impact owing to the adsorption and cooling from desorption, the CO_2_ loading and working capacities of flexible MOFs during the rapid PVSA cycle can be much larger than those of conventional adsorbents (Fig. 1c).

To demonstrate that the flexible MOFs can be breakthrough materials for actual high-throughput separation processes, the following three issues require clarification. The first involves the time constant for the gate-opening; the duration of the feed operation of a rapid PVSA process should be short (i.e., a few tens of seconds). Namely, the gate-opening accompanying the structural deformation of the host framework must respond quickly to the gas feed and be accomplished considerably faster than the cycle time. Second, the separation properties must be retained for operations under non-isothermal conditions. To the best of our knowledge, the intrinsic thermal management capability of flexible MOFs for such a condition has not been discussed. The third issue involves solving the “slipping-off” phenomenon of the flexible MOFs; flexible MOFs cannot adsorb guest molecules when the partial pressure of the guest in a gas mixture flowing in an adsorption column decreases below its specific gate-opening pressure. This has not been recognized as a major challenge in the development of gas separation systems using flexible MOFs, as it was first reported by Horike et al.^14^. However, this is a crucial matter when considering the separation for a binary mixture (*α* and *β*): using a flexible MOF that exhibits gate-opening only for component *α*, the pure component *β* cannot be obtained at the outlet of an adsorption column because of the slipping-off of component *α* from the adsorption column (Fig. 1d).

Here, we focus on ELM-11 ([Cu(BF_4_)_2_(4,4′-bipyridine)_2_]_n_)^15,16^, which has a flexible framework and shows a typical gate adsorption for CO_2_ at ambient temperatures, as an adsorbent for CO_2_/CH_4_ gas mixture separation. The separation of CO_2_/CH_4_ gas mixtures such as those found in natural gas and landfill gas have gathered attention in the field of carbon capture and storage technologies^17–19^. First, we reveal that the gate-opening/closing behaviour of ELM-11 for CO_2_ is sufficiently fast in response to the increasing/decreasing of CO_2_ gas pressure by time-resolved in situ synchrotron X-ray powder diffraction (XRPD) measurements. Second, we compare the CO_2_ separation characteristics (uptake, selectivity, working capacity, and regenerability) of ELM-11 and HKUST-1 ([Cu_3_(1,3,5-benzenetricarboxylate)_2_]_n_)^20^ having a rigid framework that was reported as the most promising adsorbent for landfill gas separation^17^. We then illustrate the superiority of ELM-11, assuming a rapid PVSA process for landfill gas separation (CO_2_:CH_4_ = 50:50) composed of the following four elementary steps: (i) pressurization and adsorption of an equimolar CO_2_/CH_4_ mixture at 500 kPa, (ii) depressurization and rinsing with pure CO_2_ at 250 kPa, (iii) desorption at 15 kPa, and (iv) purging with pure CH_4_ at 15 kPa. We finally show that the issue of the slipping-off phenomenon can be solved by improving the adsorption column from an operational point of view, and we demonstrate that our proposed rapid PVSA process using flexible MOFs is an advanced adsorption system for CO_2_ separation.

## Results

### Pressure-aided fast gating of the flexible MOF

**Fig. 2:**
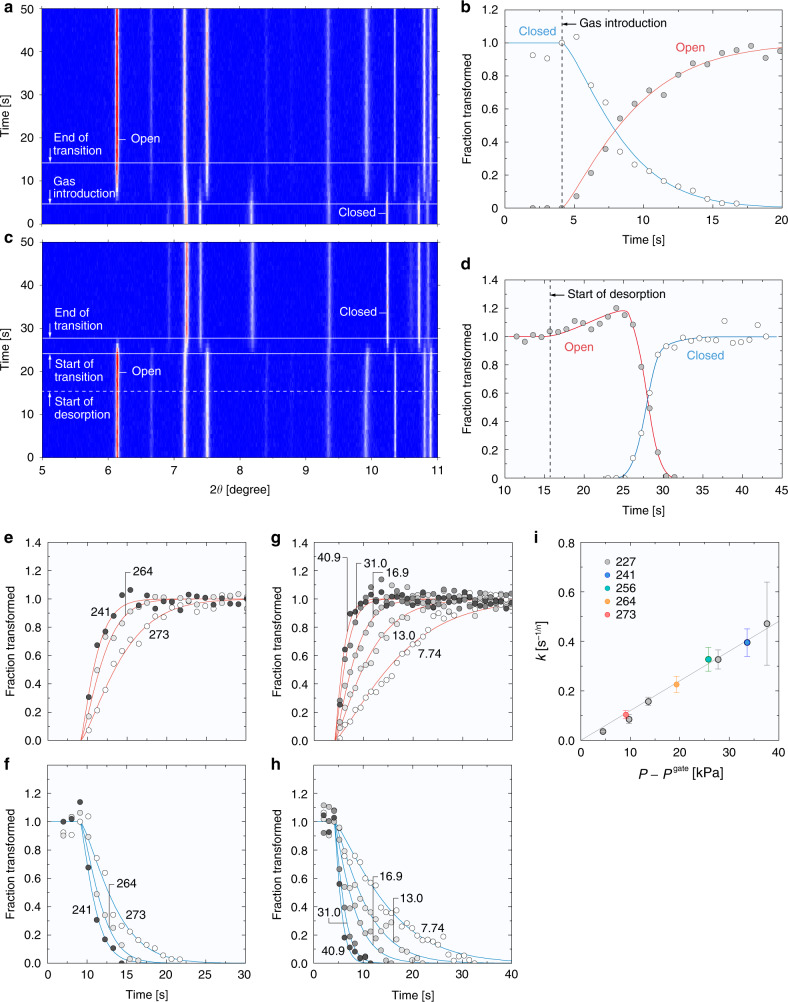
Time-resolved in situ synchrotron X-ray powder diffraction (XRPD) measurements. **a** Colour map of XRPD patterns for CO_2_ adsorption on ELM-11 at 273 K and a constant pressure of 40.8 kPa. **b** Normalized peak intensities (fractions of the phase transformed) from the 002 reflection at 6.1° for the open phase and the 020 reflection at 10.2° for the closed phase shown in **a**. The two solid lines after 4.15 s were obtained by fitting of the Kolmogorov–Johnson–Mehl–Avrami (KJMA) equation. **c** Colour map of XRPD patterns for CO_2_ desorption at 273 K during pressure depression from 100 to 3.8 kPa (the change in CO_2_ pressure is shown in Supplementary Fig. 1). **d** Fractions of the phase transformed from the 002 reflection for the open phase and the 020 reflection for the closed phase shown in **c**. **e** Fractions of the phase transformed for the open phase and **f** for the closed phase at 40.8  kPa and 241 K, at 41.0 kPa and 264 K, and at 40.8 kPa and 273 K. The numbers in **e** and **f** denote the temperature. **g** Fractions of the phase transformed for the open phase and **h** for the closed phase at 227 K as a function of the CO_2_ pressure. The numbers in **g** and **h** denote the CO_2_ gas pressure in kPa introduced. The curves after 4.15 s in **e**–**h** were obtained by fitting of the KJMA equation. **i** Relationship between the rate coefficients and the pressure difference between the CO_2_ gas pressure, *P*, and the gate-opening pressure, *P*^gate^. The error bar represents the standard deviation of the value obtained using the least-square fitting of the KJMA equation to the experimental data (*n* ≥ 3, *n*: number of experimental points used for fitting). The rate coefficients used in this plot are those for the open phase tabulated in Supplementary Table 1. The solid line in **i** was obtained from the least-squares fitting of Eq. (3).

We investigated the rate of structural transition on gating of ELM-11 by time-resolved in situ synchrotron XRPD measurements. Figure 2a,b show that the structural transition of ELM-11 started immediately after the introduction of CO_2_ at 40.8 kPa and 273 K, and was accomplished in ~10 s. We also confirmed that ELM-11 accommodating CO_2_ responded quickly to the decrease in gas pressure at 273 K: the structural transition was completed in 5 s when the CO_2_ pressure was decreased at the rate of 2.4 kPa s^−1^ (Fig. 2c,d, and Supplementary Fig. 1). Time development of the fraction of the open phase by introducing ~41 kPa of CO_2_ at 273, 264, and 241 K is shown in Fig. 2e, and that of the closed phase is shown in Fig. 2f; it can be seen that the rate of phase transition increases as the temperature decreases under the same CO_2_ pressure. We also investigated the dependence of the rate of phase transition on the pressure at 227 K, as shown in Fig. 2g,h, and found that the rate of structural transition increased with increasing CO_2_ pressure, and the phase transition was completed within a few seconds at the highest gas pressure. Furthermore, these data were found to obey the Kolmogorov–Johnson–Mehl–Avrami (KJMA) equation^21,22^, as drawn with solid lines in Fig. 2e–h. The obtained parameters are summarized in Supplementary Table 1. The KJMA parameters for the open and closed phases under the same experimental conditions are in good agreement within the fitting errors, although the errors for the closed phase are mostly larger than those for the open phase because of the lower intensity of the 020 reflection of the closed phase. Figure 2i shows the relation between the rate constant of the KJMA equation and the pressure difference, *P* − *P*^gate^, where *P* is the CO_2_ pressure introduced into the glass capillary with ELM-11 and *P*^gate^ is the gate-opening pressure. We found that a characteristic curve in proportion to *P* − *P*^gate^ could be drawn for all the rate constants of the open phase investigated. The constant of proportionality was determined to be 12.0 ± 0.5 s^−1/*n*^ MPa^−1^ by least-squares fitting of Eq. (3). This suggests that the rate constant only depends on the CO_2_ gas pressure and the temperature, which controls the gate-opening pressure, and that the gate-opening rate is amplified by the CO_2_ pressure exerted on ELM-11. This equation tells us that it takes only 1.5 s for ELM-11 to achieve 95% structural transition when CO_2_ gas of 250 kPa is introduced at 298 K. This feature is particularly suitable for the rapid PVSA proposed in the later section.

It is also worth noting that the exponents of the KJMA equation, *n*, obtained from all the data for the open phase were 1.2−1.3, which indicates the quasi-one-dimensional growth of the open phase of ELM-11^21^. This is indeed consistent with the mechanism of gate-opening predicted in the previous study:^23^ one-dimensional channels composed of the stacked two-dimensional square grid layers are formed in ELM-11 once the interlayer distance is increased, and simultaneously, the CO_2_ molecules penetrate through the one-dimensional channels (Supplementary Fig. 2), i.e. the formation of the open phase encapsulating CO_2_ proceeds along the one-dimensional direction.

### Adsorption properties under isothermal conditions

**Fig. 3:**
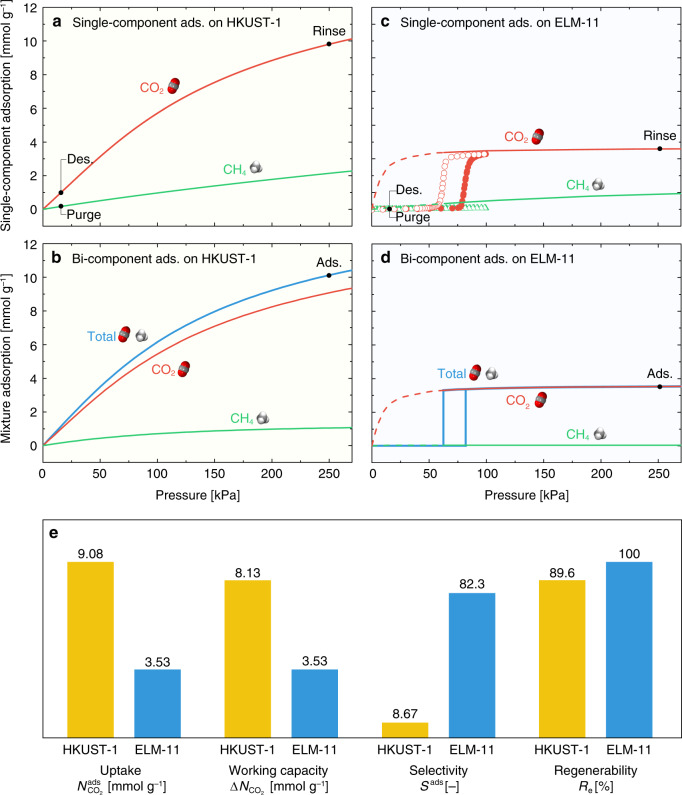
Adsorption properties of HKUST-1 and ELM-11 under isothermal conditions. Single-component adsorption isotherms of CO_2_ and CH_4_ at 298 K on **a** HKUST-1 from the experimentally determined Virial−Langmuir equations^24^ and **c** ELM-11 (filled symbols: experimental adsorption data, open symbols: experimental desorption data, lines: adsorption data simulated by grand canonical Monte Carlo (GCMC)). Total and component adsorption isotherms of an equimolar mixture of CO_2_ and CH_4_ at 298 K for **b** HKUST-1 evaluated by the ideal adsorbed solution theory^25^ and **d** ELM-11 simulated by GCMC. The abscissae of **b** and **d** correspond to the partial pressures of CO_2_. Black filled circles represent the points at which the corresponding pressures were applied in the four elementary steps of the rapid pressure vacuum swing adsorption (PVSA) process. **e** CO_2_ separation characteristics of HKUST-1 and ELM-11 assuming PVSA under isothermal conditions at 298 K.

**Fig. 4:**
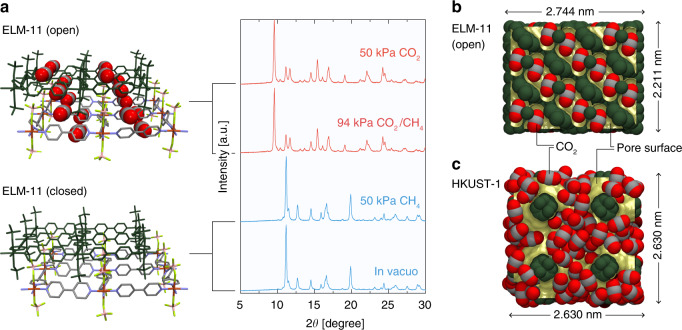
Framework structures of ELM-11 and HKUST-1. **a** In situ X-ray powder diffraction patterns (Cu Kα) of ELM-11 in vacuo and after adsorption of CO_2_ gas (50 kPa), CH_4_ gas (50 kPa), and an equimolar CO_2_/CH_4_ gas mixture (94 kPa) at 273 K together with crystal structures of ELM-11 in closed and open states determined in our previous studies^23,26^. Pore structure and CO_2_ configuration of **b** ELM-11 and **c** HKUST-1.

Figure 3a shows the experimental single-component adsorption isotherms for CO_2_ and CH_4_ on HKUST-1 at 298 K; the data were taken from the Virial–Langmuir equations experimentally determined by Chowdhury et al.^24^. The total and component adsorption isotherms of an equimolar CO_2_/CH_4_ mixture on HKUST-1 at 298 K, and the selectivity of CO_2_ over CH_4_, were evaluated by the ideal adsorbed solution theory^25^ using the experimentally obtained single-component adsorption isotherms; the obtained data are shown in Fig. 3b. Figure 3c depicts our experimentally obtained single-component adsorption isotherms for CO_2_ and CH_4_ on ELM-11 at 298 K together with their theoretical adsorption isotherms obtained by grand canonical Monte Carlo (GCMC) simulations using the quenched open framework structure of ELM-11, which was determined by the Rietveld method for pure CO_2_ gas adsorption at 298 K^23,26^. The plateau of the simulated adsorption isotherm for CO_2_ on the quenched open framework was in good agreement with that of the experimentally obtained one for CO_2_ on ELM-11, demonstrating the validity of our GCMC simulations. The fictitious GCMC isotherm for CH_4_ on the quenched open framework shows low adsorption, even though the framework has voids to accommodate CH_4_ molecules, which is due to the weak solid–fluid interactions. In contrast, the experimental isotherm of CH_4_ shows no adsorption, which is because the affinity of CH_4_ is too weak to pry open the framework of ELM-11. We also performed in situ XRPD measurements of ELM-11 in vacuo and after adsorption of CO_2_ gas (50 kPa), CH_4_ gas (50 kPa), and an equimolar mixture of CO_2_/CH_4_ gas (94 kPa) at 273 K, respectively, which are shown in Fig. 4a. The XRPD pattern of ELM-11 exposed to CH_4_ gas was the same as that in vacuo, and the XRPD pattern of ELM-11 after adsorption of the CO_2_/CH_4_ gas mixture perfectly coincided with that after gate adsorption of CO_2_. This strongly suggests that the open framework structure of ELM-11 after the adsorption of the equimolar CO_2_/CH_4_ gas mixture is the same as that after the adsorption of pure CO_2_. Hence, we computed the total and component adsorption isotherms of the equimolar CO_2_/CH_4_ gas mixture and the selectivity of CO_2_ over CH_4_ by GCMC simulations using the open framework structure of ELM-11 for pure CO_2_. The obtained results are shown in Fig. 3d.

The compatibility of the open framework structures of ELM-11 for pure CO_2_ adsorption and equimolar CO_2_/CH_4_ gas mixture adsorption can also provide insights into the slipping-off phenomenon of ELM-11. The gate-opening of ELM-11 under the flow of CO_2_/CH_4_ mixture gas should not occur when the CO_2_ partial pressure of mixture gas falls below the gate-opening pressure of ELM-11 for pure CO_2_ adsorption. Namely, the fraction of slipping-off CO_2_ in an effluent gas can be estimated as the ratio of the gate adsorption pressure for pure CO_2_ adsorption and the total gas pressure in the adsorption column. Indeed, by using our experimental gate-opening pressure of ELM-11 for pure CO_2_, we successfully predicted the effluent fraction of CO_2_ of the experimental breakthrough curve, as described in a later section.

The separation characteristics of HKUST-1 and ELM-11, evaluated by assuming that all steps of the PVSA process were operated under isothermal conditions at 298 K, are shown in Fig. 3e. The CO_2_ selectivity, *S*^ads^, of ELM-11 was 9.5 times higher than that of HKUST-1 (ELM-11: *S*^ads^ = 82.3, HKUST-1: *S*^ads^ = 8.67). Thus, the high CO_2_ selectivity of ELM-11 arises from the fact that the GCMC adsorption isotherms for the quenched open framework at low pressures (<50 kPa) show an overwhelmingly high affinity for CO_2_ over CH_4_, compared with HKUST-1. There has been an argument that the gate adsorption does not necessarily lead to high adsorption selectivity because guest molecules with low affinity should be adsorbed on the host framework pried open by other guest molecules with high affinity;^27,28^ however, this is not the case for ELM-11. Specifically, the adsorption amount of CH_4_ in the open framework is much less than that of CO_2_, as shown in Fig. 3c, even though the open framework of ELM-11 provides enough space for adsorption. This is because ELM-11 provides a purpose-built framework structure for CO_2_ in terms of size and intermolecular interactions as depicted in Fig. 4b. Owing to the large pore volume of HKUST-1 (Fig. 4c), its CO_2_ uptake, $N_{{\mathrm{CO}}_2}^{{\mathrm{ads}}}$, reached 9.08 mmol g^−1^, which is 2.6 times the CO_2_ uptake of ELM-11. After the desorption process, the amount of adsorbed CO_2_, $N_{{\mathrm{CO}}_2}^{{\mathrm{des}}}$, on HKUST-1 was 0.95 mmol g^−1^; hence, the CO_2_ working capacity of HKUST-1 decreased slightly to Δ$N_{{\mathrm{CO}}_2}$ = 8.13 mmol g^−1^, which is still 2.3 times larger than that of ELM-11. The regenerability of ELM-11 (100%) is superior to that of HKUST-1 (89.6%), which arises from the fact that the amount of CO_2_ adsorbed on ELM-11 under desorption conditions (CO_2_ pressure of 15 kPa) is zero, as a result of gate-closing. It is worth noting that if the open framework structure of ELM-11 is rigid and does not transform to the closed state, the CO_2_ adsorption isotherm during the desorption process must be type I (depicted by the red dashed line in Fig. 3c) and the Δ$N_{{\mathrm{CO}}_2}$ value is only 0.96 mmol g^−1^, which corresponds to a regenerability of 27%. In other words, for rigid adsorbents, the factors to improve regenerability and selectivity are essentially contradictory. In contrast, flexible MOFs can provide both perfect regenerability and high selectivity as long as the gate-opening and gate-closing pressures are compatible with the operating conditions of the PVSA process.

### Adsorption properties under adiabatic conditions

**Fig. 5:**
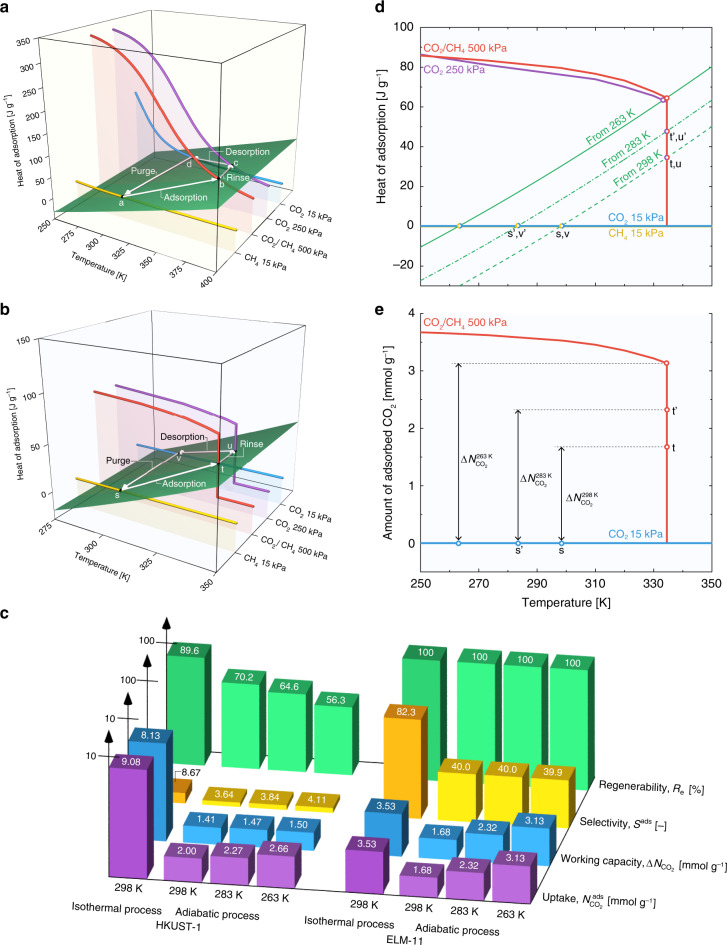
Adsorption properties of HKUST-1 and ELM-11 under adiabatic conditions. Temperature dependence of heat of adsorption for **a** HKUST-1 and **b** ELM-11. Red line: adsorption of an equimolar CO_2_/CH_4_ gas mixture at 500 kPa, purple line: pure CO_2_ adsorption at 250 kPa, blue line: pure CO_2_ adsorption at 15 kPa, and yellow line: pure CH_4_ adsorption at 15 kPa. The green plane shows the heat required to change the temperature of the host framework. White arrows represent the four adiabatic operating processes of rapid pressure vacuum swing adsorption (PVSA). The starting temperature was set as 298 K. **c** CO_2_ separation characteristics of HKUST-1 and ELM-11 for PVSA assuming the adiabatic operations and the starting temperatures of 298, 283, and 263 K together with those assuming isothermal operations at 298 K. **d** Temperature dependence of net heat of adsorption for ELM-11 for adsorption of an equimolar CO_2_/CH_4_ gas mixture at 500 kPa (red), pure CO_2_ at 250 kPa (purple), pure CO_2_ at 15 kPa (blue), and pure CH_4_ at 15 kPa (yellow). Green lines are the heat balance curves when the adiabatic adsorption process starts from 298, 283, and 263 K. **e** Temperature dependence of the amount of CO_2_ adsorbed on ELM-11 for an equimolar CO_2_/CH_4_ mixture at 500 kPa (red) and that for pure CO_2_ at 15 kPa (blue).

We evaluated the “effective adsorption properties” of HKUST-1 and ELM-11 through adiabatic separation operations, which are extreme conditions in the rapid PVSA process: the values were obtained by considering the negative effects of temperature rise/drop caused by heats of adsorption/desorption under adiabatic conditions. In this case, all of the heat generated by guest adsorption is consumed to increase the system temperature. Namely, the changes in adsorption amount and temperature in response to the pressure swing are determined such that the heat generated by adsorption is commensurate with the heat required to change the system temperature. For simplicity, the specific heat of gas can be ignored because it is much smaller than that of the adsorbent. Figure 5a depicts the heats of adsorption for HKUST-1 as a function of temperature, represented as isobars for the equimolar CO_2_/CH_4_ mixture at 500 kPa, pure CO_2_ at 250 kPa, pure CO_2_ at 15 kPa, and pure CH_4_ at 15 kPa, corresponding to the operating pressures used for the rapid PVSA process (see also Supplementary Fig. 3). The heats of adsorption and specific heat of HKUST-1 were taken from the experimental data reported by Chowdhury et al.^24^, Kloutse et al.^29^, and Mu et al^30^. The green plane in Fig. 5a represents the adiabatic operating plane calculated by integrating the specific heat of HKUST-1 with respect to temperature. Starting from point **a**, the intersection point of the green plane and yellow line (298 K), we considered performing adiabatic pressurization and adsorption with the equimolar CO_2_/CH_4_ mixture at 500 kPa. The state should move from point **a** to the intersection of the red line and the green plane (point **b**), because the heat released by adsorption and the heat absorbed by the adsorbent are equal at point **b**. This means that the temperature of HKUST-1 changes from 298 to 370 K during the adiabatic pressurization and adsorption process. If we perform adiabatic depressurization and rinsing with pure CO_2_ at 250 kPa, the state moves to point **c** (366 K), where the purple line and the green plane intersect. The adiabatic desorption of CO_2_ from 250 to 15 kPa results in a significant decrease in the system temperature, as indicated by point **d** (313 K). Finally, the state moves back to point **a** on adiabatic purging of CO_2_ with a flow of a pure CH_4_ gas at 15 kPa. The adsorption amounts of CO_2_ and CH_4_ on HKUST-1 at corresponding points **a**−**d** are listed in Table 1.

**Table 1:** Adsorbed amounts of CO_2_ and CH_4_ and temperatures at points a−d and s−v in Fig. 5a,b.

Adsorbent	Operation (point)	$N_{{\mathrm{CO}}_2}$ (mmol g^−1^)	$N_{{\mathrm{CH}}_4}$ (mmol g^−1^)	*T* (K)
HKUST-1	Adsorption (b)	2.00	0.55	370
	Rinsing (c)	2.25	0	366
	Desorption (d)	0.60	0	313
	Purging (a)	0	0.16	298
ELM-11	Adsorption (t)	1.68	0.04	334
	Rinsing (u)	1.79	0	334
	Desorption (v)	0	0	298
	Purging (s)	0	0	298

To evaluate the adsorption properties of ELM-11 under adiabatic conditions, the temperature dependences of the enthalpy change of guest −Δ*H*^guest^, the net heat *q*^net^, and the intrinsic thermal management capability *e* were evaluated. At 298 K, the exothermic heat Δ*H*^guest^ during the adsorption of the equimolar CO_2_/CH_4_ gas mixture at 500 kPa was 135.3 J g^−1^; however, on considering the endothermic heat consumed by the host expansion (Δ*H*^host^ = 55.7 J g^−1^)^13^, the net heat *q*^net^ decreased to 79.6 J g^−1^, which means that *e* = 41.1% of the exothermic heat was offset. The *e* values were always ~40% over the temperature range of 248−335 K and showed a small dependence on the temperature, demonstrating the superior intrinsic thermal management capability of ELM-11 (Supplementary Fig. 4). Figure 5b shows the obtained heats of adsorption as a function of temperature for ELM-11. The isobars for the heats of adsorption for the equimolar CO_2_/CH_4_ mixture at 500 kPa and pure CO_2_ at 250 kPa show a sharp decrease at 335 K because of the gating of ELM-11. Starting from point **s** on the green plane at 298 K, the state moves to point **t** (335 K) on adiabatic pressurization and adsorption of the equimolar CO_2_/CH_4_ mixture at 500 kPa. The green plane divides the perpendicular segment of the red line at 335 K into two parts, suggesting that 54% of ELM-11 undergoes gate adsorption (Supplementary Note 1). The state moves to point **u** upon adiabatic depressurization and rinsing with pure CO_2_ at 250 kPa; however, this process does not affect the system temperature. Adiabatic desorption of CO_2_ from 250 to 15 kPa causes the system temperature to decrease to 298 K (point **v**) and results in evacuation of all the CO_2_ molecules adsorbed on ELM-11. Therefore, purging of CO_2_ is not required for ELM-11 (point **v** → point **s**); this is an advantage of flexible MOFs showing gate adsorption. The adsorption amounts of CO_2_ and CH_4_ on ELM-11 at corresponding points **s**−**v** are also listed in Table 1.

The four separation characteristics of HKUST-1 and ELM-11 under adiabatic conditions are depicted in Fig. 5c together with those under isothermal conditions. Three data sets are classified in accordance with the “starting temperature”, which should be set for point **a** for HKUST-1 and point **s** for ELM-11. If we set the starting temperature at 298 K, the CO_2_ uptake of HKUST-1 significantly decreases to 2.00 mmol g^−1^ in contrast to 9.08 mmol g^−1^ obtained for adsorption under isothermal conditions at 298 K, becoming comparable with that of ELM-11 (1.68 mmol g^−1^) for adiabatic adsorption. The CO_2_ selectivity, *S*^ads^, also decreases for both HKUST-1 and ELM-11 because of the temperature increase of the system (Δ*T* = 71.5 K for HKUST-1 and Δ*T* = 36.5 K for ELM-11, Table 1); however, the *S*^ads^ value of ELM-11 still remains large. Furthermore, the CO_2_ working capacity of ELM-11 (1.68 mmol g^−1^) surpasses the record of HKUST-1 (1.41 mmol g^−1^).

The decrease in the starting temperature below 283 K can improve the CO_2_ uptake and working capacity of ELM-11, and all the separation characteristics of ELM-11 were found to be superior to those of HKUST-1 (Fig. 5c). This should be possible because the cyclic steady state of the PVSA process can be controlled by varying the initial temperature of the adsorption column^31^. Fig. 5d replots Fig. 5b together with the heat balance curves assuming the starting temperatures of 283 and 263 K. The PVSA cycle becomes **s**′→**t**′→**u**′→**v**′→**s**′ by setting the starting temperature as 283 K and crossing the perpendicular segment of the red line at point **t**′. The intersection point **t**′, at a higher heat of adsorption than point **t**, refers to the increase in the ratio of ELM-11 undergoing gate adsorption, wherein the CO_2_ uptake of ELM-11 is improved (Fig. 5e). If we can further lower the starting temperature to 263 K, the heat balance curve passes through the top of the perpendicular segment of the red line, and 100% of ELM-11 becomes available. This results in significant improvements in the CO_2_ uptake and working capacity of ELM-11. The starting temperature of 263 K may seem relatively low; however, some reports have shown that the temperature of adsorption columns attains values below 273 K in the cyclic steady state^32,33^. It should be also noted that, assuming that the system is completely adiabatic, the additional cost of cooling is unnecessary once the system cools down to the starting temperature and the cyclic steady state is established; hence, for a partially heat-insulated PVSA system, only a small amount of energy would be required for cooling to maintain the cyclic steady state.

In comparing the adsorption properties under the isothermal conditions and the adiabatic conditions for each adsorbent (Fig. 5c), the adsorption properties of ELM-11 are almost unaffected by the adsorption conditions, except for the CO_2_ selectivity. This fact suggests that flexible MOFs with the intrinsic thermal management capability are effective for improving the efficiency of the rapid PVSA system.

### Strategy for preventing the slipping-off problem

**Fig. 6:**
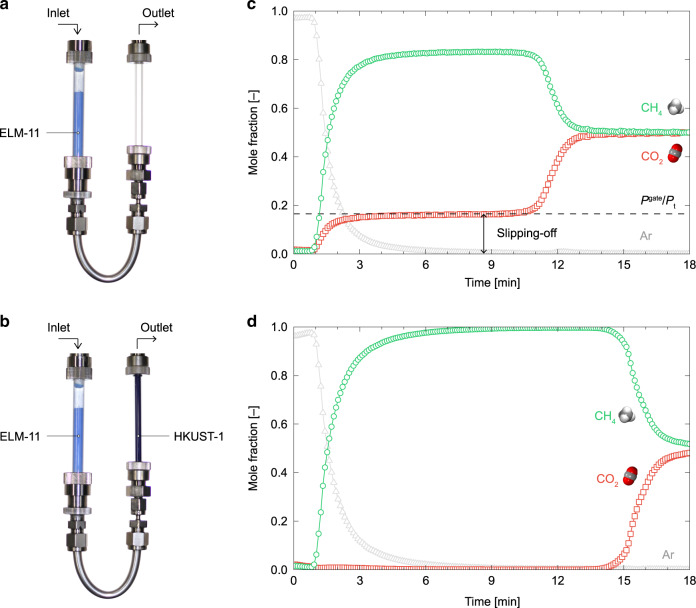
Slipping-off problem and sequential-column system as a solution to this problem. Photographs of the column system used to measure the breakthrough curves: **a** a single-column system containing ELM-11 and **b** a sequential-column system containing ELM-11 and HKUST-1. **c**, **d** are breakthrough curves for an equimolar CO_2_/CH_4_ gas mixture in **a** and **b**, respectively. The flow rate of the gas mixture was 20 sccm, and the adsorption columns were kept at 273 K. The outlet pressure was maintained at 200 kPa.

ELM-11 can exhibit superior performance in the rapid PVSA system for landfill gas separation, as described in the previous section; however, pure CH_4_ gas cannot be obtained from the system unless the slipping-off problem is solved. To show it and demonstrate our strategy for preventing the slipping-off problem, we measured the breakthrough curve of CO_2_ on ELM-11 using the two systems shown in Fig. 6a,b.

An equimolar CO_2_/CH_4_ gas mixture at 200 kPa was flowed through a column containing ELM-11 at 273 K, which was previously heat-treated and purged with pure Ar (Fig. 6a), and the gas composition of the outlet flow from the column was determined using a quadrupole mass spectrometer. As shown in Fig. 6c, the CO_2_ fraction of the outlet stream became 0.16 after extruding the preloaded Ar ~1.5 min. This is the slipping-off phenomenon, and another step around 12 min comes from the essential breakthrough of ELM-11. The fraction of slipping-off CO_2_ obtained by the breakthrough experiment is in good agreement with *P*^gate^/*P*_t_ = 0.165, where *P*^gate^ = 33.1 kPa is the gate adsorption pressure for pure CO_2_ adsorption on ELM-11 at 273 K, and *P*_t_ = 200 kPa is the total pressure of the feed gas. This result indicates that lowering the gate adsorption pressure and/or increasing the total pressure of the feed gas can reduce the molar percentage of slipping-off gas. In fact, Horike et al. performed breakthrough experiments for CO_2_/CH_4_ and CO_2_/C_2_H_6_ gas mixtures adsorbed on solid solutions of [Zn(5-nitroisophthalate)(4,4′-bipyridine)]_n_ and [Zn(5-methoxyisophthalate)(4,4′-bipyridine)]_n_. Their results demonstrated that the gate-opening pressure and the fraction of slipping-off CO_2_ was reduced by controlling the ratio of 5-nitroisophthalate/5-methoxyisophthalate^14^. Although their method is effective, it is probably applicable to only a limited range of flexible MOFs. Moreover, reducing the gating pressure would undermine the large working capacity and perfect regenerability, which are advantages of the flexible MOFs. Although over-pressurization of the feed gas was also suggested by Sotomayor and Lastoskie^34^, this would consume considerable energy because, for example, to obtain pure CH_4_ gas (99.9%), the total pressure of the feed gas should be increased to 2.08 MPa by assuming the adsorption process for an equimolar CO_2_/CH_4_ gas mixture with the starting temperature of 263 K.

**Fig. 7:**
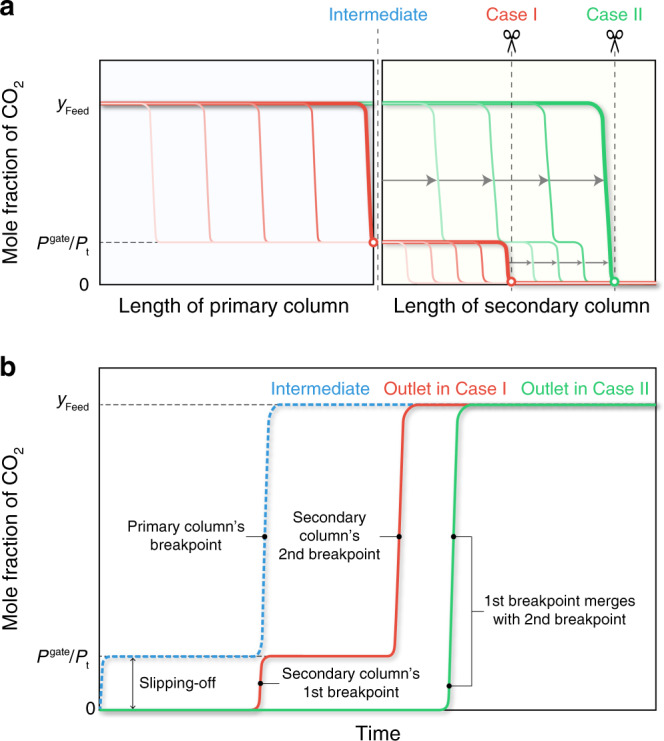
Development of CO_2_ fraction in gas stream passing through the sequential-column system. **a** Movement of the mass-transfer zone in the primary and secondary columns containing a flexible MOF and a Langmuir-type adsorbent, respectively, where red and green lines show the CO_2_ fraction in the stream in the columns before and after the primary column breaks through, respectively. **b** Breakthrough curves at the three positions indicated by dashed lines in **a**, where “intermediate” is the outlet of the primary column, “case I“ is the front of the mass-transfer zone in the secondary column when the primary column breaks through, and “case II” is the front of the mass-transfer zone when the first breakpoint just merges with the second one.

To prevent the CO_2_ slipping-off problem, we propose attaching a secondary column containing a conventional adsorbent that exhibits a Langmuir-type CO_2_ isotherm to a primary column filled with the flexible MOF. Although this system is similar to the one with multi-layered columns for separating a mixture of three or more components^35^, it is based on a new strategy using multiple items to improve the efficiency of the rapid PVSA system using the flexible MOF, as described in the following sections. To demonstrate the availability of our strategy, we set HKUST-1 in the secondary column downstream of the ELM-11 column (Fig. 6b), and we measured the breakthrough curve for the CO_2_/CH_4_ gas mixture at 200 kPa and 273 K. We successfully confirmed that no slipping-off CO_2_ was detected from the outlet of the secondary column, as shown in Fig. 6d. Note that to make the system cost-effective, the amount of conventional adsorbent for eliminating slipping-off CO_2_ can be determined, with two possible cases. Case I is to determine the amount of conventional adsorbent so that the secondary column’s breakthrough owing to the slipping-off CO_2_ matches the primary column’s breakthrough, as shown in Fig. 7a. Namely, the amount of conventional adsorbent should be the minimum quantity necessary for removing the slipping-off CO_2_, and in this case, the outlet flow from the secondary column exhibits a two-stage breakthrough, wherein the first breakpoint is related to the slipping-off CO_2_, and the second is related to the feed gas (Fig. 7b). However, it is clear that the conventional adsorbent installed in the secondary column, which was saturated by the slipping-off CO_2_, still has a redundant capacity to adsorb CO_2_. That is, after the CO_2_ fraction in the inlet flow for the secondary column was increased from the slipping-off concentration (*P*^gate^/*P*_t_) to that of the feed flow (*y*_Feed_) owing to the primary column’s breakthrough, the secondary column can be also available to remove CO_2_. Therefore, Case II should be designed to reduce the time lag between the first and second breakpoints to zero by installing a slightly larger amount of conventional adsorbent than that in Case I. This design is possible because the Langmuir-type adsorption isotherm of conventional adsorbent is convex upward (the slope of the isotherm decreases with an increasing concentration of CO_2_), and thus, the mass-transfer zone for reducing the CO_2_ fraction from *y*_Feed_ to *P*^gate^/*P*_t_ moves faster than that for reducing the CO_2_ fraction from *P*^gate^/*P*_t_ to zero (Fig. 7a). The choice between Case I and Case II should depend on the operating conditions and the characteristics of the flexible and rigid adsorbents used.

### Comparison between a conventional and our proposed system

**Fig. 8:**
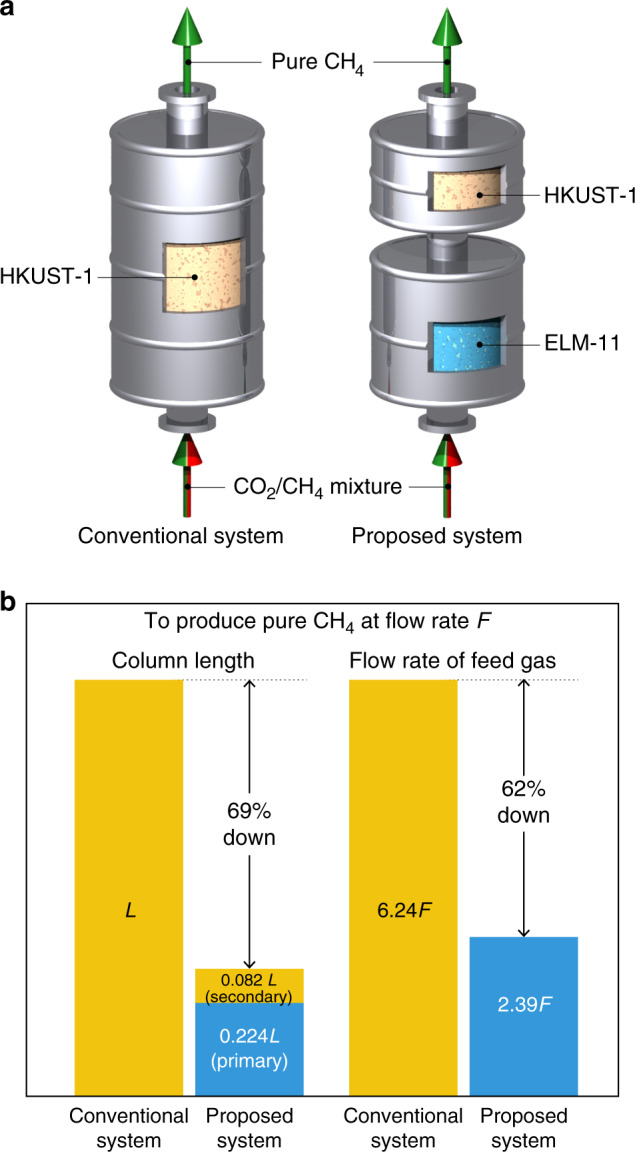
Comparison between the conventional and proposed systems. **a** Illustrations of a conventional system using HKUST-1 and our proposed system using ELM-11 and HKUST-1. **b** Comparison of column length and flow rate of feed gas required to obtain the same flow rate of product CH_4_, *F*, for the conventional system and our proposed one.

To verify the effectiveness of our proposed system, we compared the performance of two rapid PVSA processes: a conventional system using a single column filled with HKUST-1 and our proposed system where ELM-11 and HKUST-1 are filled in the primary and secondary columns, respectively. These systems are schematically illustrated in Fig. 8a. We assumed that the adiabatic adsorption of equimolar CO_2_/CH_4_ mixture at 500 kPa (starting temperature of 263 K) was performed under the same conditions with the rapid PVSA process investigated in this study. We determined the column length and the molar flow rate of the feed gas required to obtain the same molar flow rate of CH_4_ (product gas), *F*, for both systems by conducting material balance calculations using the “effective adsorption properties” under adiabatic conditions obtained in the former section. Note that the basic concept of the calculation is analogous to the screening method for the PVSA systems developed by Maring and Webley, which can provide a good estimation^36^. The obtained results are shown in Fig. 8b. When we set the size of the column of the conventional system to *L*, our proposed system based on Case II only requires a primary column with a size of 0.224*L* and a secondary column with a size of 0.082*L* to accomplish the same molar flow rate of product gas, *F*. This means that our proposed system is, overall, 69% smaller than the conventional one, even though it consists of two columns. Moreover, the molar flow rate of the feed gas for our system (2.39*F*) was also found to be 62% less than that of the conventional one (6.24*F*). These results suggest that our proposed system, using the flexible MOFs, can be more efficient than the conventional system. In addition, if both systems are set to the same size, our system can process a much larger amount of gas and thus achieve a high-throughput CO_2_ separation. Note that the proposed system based on Case I is also superior to the conventional system, as demonstrated in detail in Supplementary Note 2.

## Discussion

Time-resolved in situ synchrotron XRPD measurements revealed that the CO_2_ gate-opening of ELM-11 can be extremely fast when *P*–*P*^gate^ is large. This should be an inherent characteristics of ELM-11 originated from its framework structure because it has been reported that, conversely, other flexible MOFs exhibit slow transitions^37^. Moreover, it was found that ELM-11 accommodating CO_2_ responded quickly to the decrease in gas pressure; the gate-closing can be much faster than the gate-opening. This suggests that the desorption of CO_2_ is accelerated by the squeezing when the host framework shrinks and returns to the closed structure.

We also found that, owing to the intrinsic thermal management capability, ELM-11 has superior properties in terms of CO_2_ uptake, CO_2_ selectivity, CO_2_ working capacity, and regenerability under adiabatic operating conditions compared with those of HKUST-1, although HKUST-1 has a larger CO_2_ capacity under isothermal conditions. This suggests that the performance of flexible MOFs on a separation process operated under an adiabatic condition as a limit of the rapid PVSA process should be evaluated not only by the adsorption isotherms, but also by the net heat of adsorption, considering the intrinsic thermal management capability.

We proposed a sequential-column system to solve the problem of the slipping-off phenomenon with flexible MOFs and demonstrated the availability of it by measuring breakthrough curves. The proposed system with ELM-11 and HKUST-1, operating under adiabatic conditions, can produce the same amount of CH_4_ with much smaller columns and less feed than those of the conventional single-column system using HKUST-1. It is worth noting that our proposed sequential-column system is suitable for flexible MOFs showing pressure-aided fast gating because the primary column containing a flexible MOF can be designed to treat a gas mixture with a high concentration of CO_2_ or a large *P* − *P*^gate^ value. The secondary column should therefore be designed to process the slipping-off CO_2_ and to maximize the utilization of fast gating of the flexible MOFs. Further, note that filling a simple mixture of a flexible MOF and a rigid nanoporous material into one adsorption column cannot inherently solve the slipping-off problem. Mixing the materials only produces a type IV isotherm (IUPAC classification^38^) as a sum of the gate adsorption and Langmuir-type isotherms, which cannot avoid the two-stage breakthrough of CO_2_, although a modest amount of pure CH_4_ can be obtained before the first breakthrough of CO_2_ (see Supplementary Fig. 5 for more details). The efficiency of the sequential-column system can be further improved if the operations for the primary column filled with a flexible MOF and the secondary column filled with a conventional adsorbent are optimized and performed individually. This is technically feasible by installing a multi-way valve at the junction of the two columns, which enables simultaneous operations of the desorption process for the primary column and purging process for the secondary column, leading to a reduction in the total cycle time (Supplementary Fig. 6). Thus, our sequential-column system can provide a variety of options for the rapid PVSA operations. Our system is also highly customizable for other separation applications because the resolution of the slipping-off problem of flexible MOFs enables effective separation of a mixture of gases, one or both of which have high added value, such as C_3_H_6_/C_3_H_8_, C_2_H_4_/C_2_H_6_, and H_2_/CH_4_. Accordingly, we believe that our methodology provides a significant advance in the field of PVSA using solid adsorbents. Moreover, we anticipate that the intrinsic thermal management and the pressure-aided fast gating capabilities of flexible MOFs will also be useful for increasing the efficiency of TSA for CO_2_ capture, which has been comprehensively studied by Hefti et al.^39^.

It should be also noted that, to realize rapid PVSA using the flexible MOFs, some issues must be resolved, for example, the hindrance of gate-opening by the pelletization of flexible MOFs, pellets braking owing to the gate-opening, and a pressure drop owing to the volume expansion of the pellets induced by the gate-opening. We have already started addressing these concerns, and the results will be published elsewhere.

## Methods

### Rapid PVSA process for landfill gas separation

We considered the following four elementary steps for the rapid PVSA process: (i) co-current pressurization and adsorption of an equimolar CO_2_/CH_4_ mixture at 500 kPa, (ii) co-current depressurization and rinsing with pure CO_2_ at 250 kPa, (iii) counter-current desorption at 15 kPa (a bench-scale PVSA plant for CO_2_ separation using NaX zeolite is operated at 10 kPa^40^), and (iv) counter-current purging of remaining CO_2_ in the adsorbent with pure CH_4_ at 15 kPa.

For simplicity, we assumed that the heat transfer from the adsorbent to the mixture gas and the adsorption tower can be ignored, and that the heat transfer between the adsorbent crystals is sufficiently rapid, which should correspond to the conditions under rapid and adiabatic adsorption/desorption operations.

The following four factors were investigated to evaluate the CO_2_ separation characteristics of ELM-11 and HKUST-1:

1. CO_2_ uptake: $$N_{{\mathrm{CO}}_2}^{{\mathrm{ads}}}$$,
2. CO_2_ selectivity: $$S^{{\mathrm{ads}}} = (N_{{\mathrm{CO}}_2}^{{\mathrm{ads}}}/N_{{\mathrm{CH}}_4}^{{\mathrm{ads}}})/(y_{{\mathrm{CO}}_2}/y_{{\mathrm{CH}}_4})$$,
3. CO_2_ working capacity: ${\mathrm{{\Delta}}}N_{{\mathrm{CO}}_2} = N_{{\mathrm{CO}}_2}^{{\mathrm{ads}}} - N_{{\mathrm{CO}}_2}^{{\mathrm{des}}}$, and
4. regenerability:$$R_{\mathrm{e}} = {\mathrm{{\Delta}}}N_{{\mathrm{CO}}_2}/N_{{\mathrm{CO}}_2}^{{\mathrm{ads}}}$$,

where *N*_*i*_ is the amount of component *i* adsorbed, *y*_*i*_ is the mole fraction of component *i* in the gas phase, and superscripts ‘ads’ and ‘des’ denote adsorption and desorption operating conditions, respectively.

### CO_2_ and CH_4_ adsorption measurements

Pre-ELM-11 was purchased from Tokyo Chemical Industry Co. and was transformed into ELM-11 by heating at 373 K for 10 h under a vacuum (< 0.1 mPa). The adsorption isotherms of CO_2_ over the temperature range of 195–298 K and CH_4_ at 298 K on ELM-11 were obtained using a BELSORP-max (MicrotracBEL Co.) and a cryostat equipped with a two-stage Gifford–McMahon (GM) refrigerator^41^. The sample cell temperature fluctuation was maintained within ±0.01 K during the adsorption measurements.

### In situ XRPD measurements

The in situ XRPD patterns of ELM-11 in vacuo and after adsorption of CO_2_ gas (50 kPa), CH_4_ gas (50 kPa), and an equimolar CO_2_/CH_4_ mixture gas (94 kPa) at 273 K were obtained using a SmartLab instrument with Cu Kα radiation (Rigaku Co.), which has a cryostat equipped with a single-stage GM refrigerator connected to BELSORP-18 volumetric adsorption equipment (MicrotracBEL Co.). ELM-11 was transformed from pre-ELM-11 under the same conditions mentioned above, and the sample cell temperature was kept within ±0.01 K during the XRPD measurements.

### Time-resolved in situ synchrotron XRPD measurements

The pre-ELM-11 sample was placed at the end of a 0.3-mm-diameter borosilicate glass capillary, which was attached to a stainless-steel tube with an epoxy adhesive. The sample was then evacuated for 10 h at 373 K to transform it into ELM-11. Time-resolved in situ synchrotron XRPD patterns of ELM-11 during gate adsorption of CO_2_ at 273 K were measured on the BL02B2 beamline of the SPring-8 synchrotron facility, Japan, using a large Debye-Scherrer-type diffractometer with a multi-modular system constructed with six MYTHEN detectors^42,43^. The temperature of the glass capillary was controlled by a nitrogen gas blower at 273 K, and the wavelength of the incident X-rays was 0.099899 nm. The in situ synchrotron XRPD patterns were continuously obtained by exposing the sample for 1 s at regular intervals of 50 ms. After 4.15 s from starting the XRPD measurements, CO_2_ gas at 103 kPa filled in a gas manifold of a remote gas handling system was automatically introduced into the glass capillary in which the ELM-11 sample was maintained in a vacuum. The CO_2_ gas pressure was decreased to 41 kPa within 1 s at the latest and the pressure was rigorously kept constant during the XRPD measurements. In the case of desorption measurement, the CO_2_ gas was rapidly depressed from 100 to 3.8 kPa through a needle valve; the XRPD measurements were performed under the same conditions for the adsorption measurement. Peak intensities of the 020 reflection at 10.2° for the closed phase of ELM-11 and the 002 reflection at 6.1° for the open phase of ELM-11 were obtained by nonlinear least-squares fitting of the pseudo-Voigt function. The fractions of each phase transformed as a function of time were determined by normalizing the peak intensities by those after completion of the transition for the open phase and before gas introduction for the closed phase. For the adsorption process, the following KJMA equation^21,22^ was fitted to the relation between the fraction transformed of the open phase and time:

1 $$\alpha = 1 - {\mathrm{exp}}\left( { - kt^n} \right),$$

where *α* is the fraction transformed at time *t*, *k* is the rate constant, and *n* is the number of dimensions in which the transition occurs. For the closed phase, the equation is arranged as

2 $$\alpha = {\mathrm{exp}}\left( { - kt^n} \right).$$

The fitting of the KJMA equation was performed for the data after the introduction of CO_2_ gas.

Time-resolved in situ synchrotron XRPD measurements for the adsorption process on ELM-11 were also performed at 264 K for the CO_2_ pressure of 41.0 kPa, at 256 K for 40.8 kPa, at 241 K for 40.8 kPa, and at 227 K for the five constant CO_2_ pressures of 7.74, 13.0, 16.9, 31.0, and 40.8 kPa. The pressure of CO_2_, *P*, introduced into the glass capillary was controlled by preliminarily adjusting the pressure of CO_2_ dosed in the gas manifold of the remote gas handling system. The correlation between *k*, and the pressure difference between *P* and the gate-opening pressure *P*^gate^ was investigated for all the open phase data. This is formulated as follows:

3 $$k\left( {P,T} \right) = k_0\left( {P - P^{{\mathrm{gate}}}} \right),$$

where *k*_0_ is a constant and the gate-opening pressure at the corresponding temperature *T* is evaluated by the relation obtained from the experimental adsorption isotherms over a wide temperature range of 195–298 K (Supplementary Fig. 7):

4 $${\mathrm{ln}}P^{{\mathrm{gate}}}\left[ {{\mathrm{kPa}}} \right] = - 3064.5/T\left[ {\mathrm{K}} \right] + 14.683.$$

### GCMC simulations

Adsorption isotherms of CO_2_ and CH_4_ on ELM-11 with the open framework structure were obtained using our own GCMC code. The Rietveld refined structure of ELM-11 at 298 K, as determined in our previous work^23^, was used as the open framework structure. The framework atoms were fixed, and four trial moves (displacement, rotation, creation, and deletion) for CO_2_ and three trial moves (displacement, creation, and deletion) for CH_4_ were performed with the equal probabilities. The system was equilibrated at 1 × 10^7^ Monte Carlo steps, after which data were collected for another 1 × 10^7^ steps. The length of the Markov chain of 1 × 10^7^ steps corresponds to >6 × 10^4^ trials per guest molecule. The simulation box was constructed with 3 × 3 × 2 unit cells and periodic boundary conditions were applied in the *a*, *b*, and *c* directions.

The guest–guest and guest–host interaction potential, *U*_guest_, was assumed to be the sum of the Coulombic and Lennard-Jones (LJ) potentials:

5 $$U_{{\mathrm{guest}}} = U_{{\mathrm{Coulombic}}} + U_{{\mathrm{LJ}}},$$

6 $$U_{{\mathrm{Coulombic}}} = {\sum} {\frac{{q_iq_j}}{{4\pi \varepsilon _0r_{ij}}}} ,$$

7 $$U_{{\mathrm{LJ}}} = {\sum} 4 \varepsilon _{ij}\left[ {\left( {\frac{{\sigma _{ij}}}{{r_{ij}}}} \right)^{12} - \left( {\frac{{\sigma _{ij}}}{{r_{ij}}}} \right)^6} \right],$$

where *q*_*i*_ is the atomic charge, *ε*_0_ (8.8542 × 10^–12^ C^2^ N^–1^ m^–2^) is the permittivity in vacuum, *r*_*ij*_ is the interatomic distance, and *σ*_*ij*_ and *ε*_*ij*_ are the LJ parameters. The Ewald summation method was used to correct the long-range Coulombic interactions with a charge screening constant of 2.0 nm^–1^ and reciprocal space sum for ***k*** vectors of *L*_*a*_*/2π*|***k***|, *L*_*b*_*/2π*|***k***|, and *L*_*c*_*/2π*|***k***|< 10 (where *L*_*a*_, *L*_*b*_, and *L*_*c*_ are the dimensions of the simulation box). The Lorentz-Berthelot mixing rules were applied to calculate the short-range interactions and were truncated at a cutoff distance of 1.6289 and 1.5557 nm for CO_2_ and CH_4_, respectively. The partial atomic charges of the host framework were obtained using periodic density functional theory (DFT) calculations at the GGA-PBE/DNP level and Mulliken population analysis using the DMol^3^ package^44,45^. The universal force field (UFF)^46^ was applied to calculate the LJ interaction term of the framework atoms by modifying the energy parameters as 0.74*ε*_UFF_^26^. The atomic coordinates, charges, and LJ parameters of the host framework are listed in Supplementary Tables 2 and 3. The interaction parameters for CO_2_^47^ and CH_4_^48^ are listed in Supplementary Tables 4 and 5.

The adsorption isotherms for an equimolar CO_2_/CH_4_ gas mixture on ELM-11 were obtained by using the RASPA^49^ software. We used the same settings and force fields for ELM-11, CO_2_, and CH_4_, as mentioned above.

### Specific heat of ELM-11 and HKUST-1

To evaluate the specific heat of ELM-11, we conducted phonon calculations for the closed and open states of ELM-11 according to the density functional perturbation theory using the VASP^50,51^ and PHONOPY^52^ codes. For the calculations using VASP, we implemented projector-augmented wave method pseudopotentials^53^ with the GGA-PBE exchange-correlation functional and 400 eV plane wave cutoffs. We used a 1 ×  1 × 1 unit cell (two primitive cells) for both the states because of the large cell size (204 and 228 atoms for the closed and open structures, respectively). The Brillouin zone of the closed state was sampled using a 3 × 3 × 2 Gamma-centred Monkhorst-Pack *k*-point mesh, and that of the open state was sampled using a 2 × 3 × 2 *k*-point mesh.

When the lattice vibrations are described by the harmonic approximation, the specific heat at constant volume, *C*_v_, can be represented as

8 $$C_{\mathrm{V}} = \mathop {\sum }\limits_{{\mathbf{q}},\nu } k_{\mathrm{B}}\left( {\frac{{\hbar \omega \left( {{\mathbf{q}},\nu } \right)}}{{k_{\mathrm{B}}T}}} \right)^2\frac{{\exp \left( {\frac{{\hbar \omega \left( {{\mathbf{q}},\nu } \right)}}{{k_{\mathrm{B}}T}}} \right)}}{{\left[ {\exp \left( {\frac{{\hbar \omega \left( {{\mathbf{q}},\nu } \right)}}{{k_{\mathrm{B}}T}}} \right) - 1} \right]^2}},$$

where **q** is the wave vector, *ν* is the index for the phonon branch, *k*_B_ is the Boltzmann factor, *ħ* is the reduced Planck constant, *ω* is the vibrational frequency, and *T* is the temperature. Here, the specific heat at constant pressure, *C*_p_, was assumed to be equal to *C*_v_ because we assumed adsorption and desorption processes under moderate temperatures. The summation for **q** and *ν* implies the addition for all the vibrational frequencies in the phonon density of states. The obtained phonon density of states and specific heat of ELM-11 as a function of temperature are shown in Supplementary Figs 8 and 9, respectively. The regular specific heat of ELM-11 in the open state at 298 K, obtained using this calculation, was 0.94 J g^−1^ K^−1^.

We fitted a polynomial function to the experimental specific heats of HKUST-1 reported by Kloutse et al.^29^ (below 320 K) and Mu et al.^30^ (over 333 K), and the function was integrated with respect to temperature to evaluate the heat required to change the temperature of the host framework (green plane in Fig. 5a). The parameters of the polynomial function are listed in Supplementary Table 6, and the specific heat of HKUST-1 as a function of temperature is shown in Supplementary Fig. 9. The regular specific heat of HKUST-1 at 298 K was 0.78 J g^−1^ K^−1^.

### Transition enthalpy and thermal management capability

The transition enthalpy during gate adsorption, required in order to assess the intrinsic thermal management capability, is expressed as:^13^

9 $${\mathrm{{\Delta}}}H^{{\mathrm{trs}}} = {\mathrm{{\Delta}}}H^{{\mathrm{host}}} + {\mathrm{{\Delta}}}H^{{\mathrm{guest}}}\left( {P^{{\mathrm{gate}}}} \right) = {\mathrm{{\Delta}}}H^{{\mathrm{host}}} - N_{{\mathrm{op}}}^{{\mathrm{guest}}}\left( {P^{{\mathrm{gate}}}} \right)q_{{\mathrm{op}}}^{{\mathrm{int}}}\left( {P^{{\mathrm{gate}}}} \right),$$

where Δ*H*^host^ and Δ*H*^guest^(*P*^gate^) are the enthalpy changes of the host and guest during gate adsorption, and $N_{{\mathrm{op}}}^{{\mathrm{guest}}}\left( {P^{{\mathrm{gate}}}} \right)$ and $q_{{\mathrm{op}}}^{{\mathrm{int}}}\left( {P^{{\mathrm{gate}}}} \right)$ are the amount of gas adsorbed on the open framework and the molar integral heat at the gate pressure *P*^gate^, respectively. Here, the enthalpies are given as values per weight of adsorbent. $q_{{\mathrm{op}}}^{{\mathrm{int}}}$ is positive when heat is released from the system. When the pressure is increased to *P* after the gate-opening at *P*^gate^ and additional guest adsorption occurs without host deformation, the heat of adsorption, *q*^add^, is released from the system:

10 $$q^{{\mathrm{add}}}\left( P \right) = N_{{\mathrm{op}}}^{{\mathrm{guest}}}\left( P \right)q_{{\mathrm{op}}}^{{\mathrm{int}}}\left( P \right) - N_{{\mathrm{op}}}^{{\mathrm{guest}}}\left( {P^{{\mathrm{gate}}}} \right)q_{{\mathrm{op}}}^{{\mathrm{int}}}\left( {P^{{\mathrm{gate}}}} \right).$$

Hence, the net heat, *q*^net^, released by the adsorption through the gate-opening at *P* (>*P*^gate^) is written as

11 $$q^{{\mathrm{net}}}\left( P \right) = - {\mathrm{{\Delta}}}H^{{\mathrm{trs}}} + q^{{\mathrm{add}}}\left( P \right) = N_{{\mathrm{op}}}^{{\mathrm{guest}}}\left( P \right)q_{{\mathrm{op}}}^{{\mathrm{int}}}\left( P \right) - {\mathrm{{\Delta}}}H^{{\mathrm{host}}}.$$

$q_{{\mathrm{op}}}^{{\mathrm{int}}}\left( P \right)$ can be evaluated using the following equation:

12 $$q_{{\mathrm{op}}}^{{\mathrm{int}}}\left( P \right) = - \frac{{H_{{\mathrm{op}}}^{{\mathrm{guest}}}\left( P \right) - H^{{\mathrm{gas}}}\left( P \right)}}{{N_{{\mathrm{op}}}^{{\mathrm{guest}}}\left( P \right)}} \approx u_{{\mathrm{op}}}^{{\mathrm{guest}}}\left( P \right) + RT,$$

where $H_{{\mathrm{op}}}^{{\mathrm{guest}}}\left( P \right) - H^{{\mathrm{gas}}}\left( P \right)$ is the enthalpy change when $N_{{\mathrm{op}}}^{{\mathrm{guest}}}\left( P \right)$ of the gas molecules are encapsulated in the open framework, $u_{{\mathrm{op}}}^{{\mathrm{guest}}}\left( P \right)$ is the molar interaction potential energy of the guest molecules, *R* is the gas constant, and *T* is the temperature. We performed GCMC simulations for the open framework to obtain the values of $N_{{\mathrm{op}}}^{{\mathrm{guest}}}\left( P \right)$ and $u_{{\mathrm{op}}}^{{\mathrm{guest}}}\left( P \right)$. We then assumed Δ*H*^host^ = Δ*U*^host^ + *P*Δ*V* ≈ Δ*U*^host^, where Δ*V* is the volume change of the host and Δ*U*^host^ is the internal energy change of the host because the *P*Δ*V* term is negligible compared to Δ*U*^host^ in most cases. We define the intrinsic thermal management capability, *e*, as

13 $$e = \frac{{{\mathrm{{\Delta}}}H^{{\mathrm{guest}}}\left( P \right) - q^{{\mathrm{net}}}\left( P \right)}}{{{\mathrm{{\Delta}}}H^{{\mathrm{guest}}}\left( P \right)}},$$

which represents the ratio of the endothermic heat caused by host deformation to the total exothermic heat resulting from guest desorption.

### Adsorption amounts and heat of adsorption on HKUST-1

The amounts of CO_2_ and CH_4_ adsorbed on HKUST-1 were evaluated using the Virial–Langmuir equation reported by Chowdhury et al.^24^:

14 $$f 	= {\frac{{n^{{\mathrm{max}}}\left( T \right)n(f,T)}}{{\beta \left( T \right)\left\{ {n^{{\mathrm{max}}}\left( T \right) - n\left( {f,T} \right)} \right\}}}\exp \left\{ {b\left( T \right)n\left( {f,T} \right) + c\left( T \right)n\left( {f,T} \right)^2} \right\}} \\ {n^{{\mathrm{max}}}\left( T \right)} 	= {\eta _0 + \frac{{\eta _1}}{T}} \\ {\beta \left( T \right)} 	= {\beta _0\exp \left( {\frac{{\beta _1}}{T}} \right)} \\ {b\left( T \right)} 	= {b_0 + \frac{{b_1}}{T}} \\ {c\left( T \right)} 	= {c_0 + \frac{{c_1}}{T},}$$

where *f* is the fugacity, *n* is the amount adsorbed, and *η*_*i*_, *β*_*i*_, *b*_*i*_, and *c*_*i*_ (*i* = 0, 1) are parameters that are listed in Supplementary Table 7. We assumed that the fugacity can be replaced by the pressure of a real gas because we treat the adsorption and desorption processes only under moderate pressures and temperatures. The isosteric heat of adsorption, *q*^st^, can be obtained by substituting Eq. (14) into the Clausius–Clapeyron equation:

15 $$ - \frac{{q^{{\mathrm{st}}}\left( {n,T} \right)}}{R} 	= \left( {\frac{{\partial {\mathrm{ln}}P}}{{\partial \left( {1/T} \right)}}} \right)_n\\ 	= - \beta _1 + b_1n\left( T \right) + c_1n\left( T \right)^2 + \frac{{\eta _1}}{{n^{{\mathrm{max}}}\left( T \right)}} - \frac{{\eta _1}}{{n^{{\mathrm{max}}}\left( T \right) - n\left( T \right)}}.$$

The total heat of adsorption *Q*, as depicted in Fig. 5a, was obtained using

16 $$Q = \mathop {\smallint }\limits_0^{n_{{\mathrm{CO}}_2}} q_{{\mathrm{CO}}_2}^{{\mathrm{st}}}{\mathrm{d}}n_{{\mathrm{CO}}_2} + \mathop {\smallint }\limits_0^{n_{{\mathrm{CH}}_4}} q_{{\mathrm{CH}}_4}^{{\mathrm{st}}}{\mathrm{d}}n_{{\mathrm{CH}}_4}.$$

### Adsorption isobars and heat of adsorption on ELM-11

It has been reported that the logarithms of the gate opening and closing pressures of flexible MOFs should be proportional to the reciprocal temperature^54^, and we confirmed that the gate opening and closing pressures of CO_2_ on ELM-11 also followed the relationship shown in Supplementary Fig. 7. Therefore, by using the ln*P*^gate^ vs reciprocal temperature plot, we evaluated the theoretical adsorption isobars and heats of adsorption on ELM-11 for an equimolar CO_2_/CH_4_ gas mixture at 500 kPa, pure CO_2_ at 250 kPa, pure CO_2_ at 15 kPa, and pure CH_4_ at 15 kPa, which correspond to the operating pressures used for the adsorption, rinsing, desorption, and purging processes, respectively. First, we determined the gate-opening/-closing temperatures of ELM-11 for pure CO_2_ as 335 K/341 K at 250 kPa and 256 K/262 K at 15 kPa, respectively, from Supplementary Fig. 7. Then, we assumed that the gate-opening/-closing temperatures for the equimolar CO_2_/CH_4_ gas mixture at 500 kPa were the same as those for pure CO_2_ at 250 kPa, which was based on the fact that the molar fraction of slipping-off CO_2_ could be predicted using the gate-opening pressure for pure CO_2_, as shown in Fig. 6c. Finally, we calculated the adsorption amounts of CO_2_ and CH_4_ on the open framework structure of ELM-11 and the corresponding heats of adsorption at temperatures below the gate-opening temperature using the GCMC simulations. The adsorption loadings of CO_2_ and CH_4_ and the corresponding heats of adsorption at temperatures above the gate-opening temperature were set to be zero because the closed framework structure showed no adsorption of CO_2_ and CH_4_.

### Breakthrough curve measurement

Breakthrough curves were measured using lab-made equipment consisting of three mass-flow controllers (HORIBA STEC Co.), a static gas mixer, a ∅ 9 mm glass tube as the primary column, a ∅ 6 mm glass tube as the secondary column, a bypass flow path, a back-pressure regulator, and a quadrupole mass spectrometer (CANON ANELVA Co.). The flowsheet of the apparatus is illustrated in Supplementary Fig. 10. The pre-ELM-11 sample used in this experiment was synthesized using the protocol reported by Kondo et al.^16^ after modification: a methanol solution of 4,4′-bipyridine (1.60 m, 365 mL) was added to a water (200 mL)-methanol (92 mL) mixed solution of Cu(II) tetrafluoroborate (1.00 m) for 2 h with vigorous stirring at room temperature. After additional stirring (1 h), the reaction mixture was filtrated, and the filter cake was washed with water three times. The blue powder was dried at room temperature under a vacuum below 5 mPa for 12 h. HKUST-1 (BasoliteC 300) was purchased from Sigma-Aldrich. Pre-ELM-11 and HKUST-1 were placed in the adsorption columns and activated by heating at 393 K for 12 h under a vacuum (< 0.1 mPa). The adsorption columns were then purged with 200 kPa of pure Ar and kept at 273 K using a thermostatic bath. Next, an equimolar CO_2_/CH_4_ gas mixture was flowed through the bypass flow path. Its flow rate was controlled to be 20 sccm (standard cm^3^ min^−1^) by the mass-flow controllers, and the system pressure was maintained at 200 kPa by the back-pressure regulator. Then, the feed stream was switched from the bypass channel to the adsorption columns side, and the gas composition downstream of the back-pressure regulator was measured using the mass spectrometer, which was calibrated using certified CO_2_/CH_4_/Ar gas mixtures before measuring the breakthrough curves.

### Material balance calculations for column systems

We evaluated the column length and molar flow rate of the feed gas required to obtain the same molar flow rate of CH_4_ for conventional and sequential-column systems, including case I and case II, via material balance calculations. The details of the material balance calculations are provided in the Supplementary Method.

## Supplementary information

Supplementary Information

Peer Review File

## Data Availability

The data that support the findings of this study are provided in Supplementary Information and the online repository at https://github.com/2koza/slipping-off. They can also be available from the corresponding author upon request.

## Code availability

The codes used in this study are available from the corresponding author upon reasonable request.

## References

[1] Sholl DS, Lively RP (2016). Seven chemical separations to change the world. Nature.

[2] Lopes FVS, Grande CA, Rodrigues AE (2012). Fast-cycling VPSA for hydrogen purification. Fuel.

[3] Sircar S, Hanley BF (1995). Production of oxygen enriched air by rapid pressure swing adsorption. Adsorption.

[4] Chai SW, Kothare MV, Sircar S (2011). Rapid pressure swing adsorption for reduction of bed size factor of a medical oxygen concentrator. Ind. Eng. Chem. Res..

[5] Horstmeier JF, Gomez Lopez A, Agar DW (2016). Performance improvement of vacuum swing adsorption processes for CO_2_ removal with integrated phase change material. Int. J. Greenh. Gas. Con..

[6] Schneemann A (2014). Flexible metal–organic frameworks. Chem. Soc. Rev..

[7] Horike S, Shimomura S, Kitagawa S (2009). Soft porous crystals. Nat. Chem..

[8] Fairen-Jimenez D (2011). Opening the gate: framework flexibility in ZIF-8 explored by experiments and simulations. J. Am. Chem. Soc..

[9] Coudert F-X, Boutin A, Fuchs AH, Neimark AV (2013). Adsorption deformation and structural transitions in metal–organic frameworks: from the unit cell to the crystal. J. Phys. Chem. Lett..

[10] Krause S (2016). A pressure-amplifying framework material with negative gas adsorption transitions. Nature.

[11] Verploegh RJ, Nair S, Sholl DS (2015). Temperature and loading-dependent diffusion of light hydrocarbons in ZIF-8 as predicted through fully flexible molecular simulations. J. Am. Chem. Soc..

[12] Mason JA (2015). Methane storage in flexible metal–organic frameworks with intrinsic thermal management. Nature.

[13] Hiraide S, Tanaka H, Ishikawa N, Miyahara MT (2017). Intrinsic thermal management capabilities of flexible metal–organic frameworks for carbon dioxide separation and capture. ACS Appl. Mater. Interfaces.

[14] Horike S, Inubushi Y, Hori T, Fukushima T, Kitagawa S (2012). A solid solution approach to 2D coordination polymers for CH_4_/CO_2_ and CH_4_/C_2_H_6_ gas separation: equilibrium and kinetic studies. Chem. Sci..

[15] Li D, Kaneko K (2001). Hydrogen bond-regulated microporous nature of copper complex-assembled microcrystals. Chem. Phys. Lett..

[16] Kondo A (2006). Novel expansion/shrinkage modulation of 2D layered MOF triggered by clathrate formation with CO_2_ molecules. Nano Lett..

[17] Bae YS, Snurr RQ (2011). Development and evaluation of porous materials for carbon dioxide separation and capture. Angew. Chem. Int. Ed..

[18] Kenarsari SD (2013). Review of recent advances in carbon dioxide separation and capture. RSC Adv..

[19] Choi S, Drese JH, Jones CW (2009). Adsorbent materials for carbon dioxide capture from large anthropogenic point sources. ChemSusChem.

[20] Chui SS (1999). A chemically functionalizable nanoporous material [Cu_3_(TMA)_2_(H_2_O)_3_]_n_. Science.

[21] De Bruijn TJW, De Jong WA, Van Den Berg PJ (1981). Kinetic parameters in Avrami—Erofeev type reactions from isothermal and non-isothermal experiments. Thermochim. Acta.

[22] Krüger P (1993). On the relation between non-isothermal and isothermal Kolmogorov-Johnson-Mehl-Avrami crystallization kinetics. J. Phys. Chem. Solids.

[23] Hiraide S, Tanaka H, Miyahara MT (2016). Understanding gate adsorption behaviour of CO_2_ on elastic layer-structured metal–organic framework-11. Dalton Trans..

[24] Chowdhury P, Mekala S, Dreisbach F, Gumma S (2012). Adsorption of CO, CO_2_ and CH_4_ on Cu-BTC and MIL-101 metal organic frameworks: Effect of open metal sites and adsorbate polarity. Micropor. Mesopor. Mater..

[25] Myers AL, Prausnitz JM (1965). Thermodynamics of mixed-gas adsorption. AlChE J..

[26] Tanaka H, Hiraide S, Kondo A, Miyahara MT (2015). Modeling and visualization of CO_2_ adsorption on elastic layer-structured metal–organic framework-11: Toward a better understanding of gate adsorption behavior. J. Phys. Chem. C..

[27] D’Alessandro DM, Smit B, Long JR (2010). Carbon dioxide capture: prospects for new materials. Angew. Chem. Int. Ed..

[28] Pirngruber GD (2012). A method for screening the potential of MOFs as CO_2_ adsorbents in pressure swing adsorption processes. ChemSusChem.

[29] Kloutse FA, Zacharia R, Cossement D, Chahine R (2015). Specific heat capacities of MOF-5, Cu-BTC, Fe-BTC, MOF-177 and MIL-53 (Al) over wide temperature ranges: Measurements and application of empirical group contribution method. Micropor. Mesopor. Mater..

[30] Mu B, Walton KS (2011). Thermal analysis and heat capacity study of metal-organic frameworks. J. Phys. Chem. C..

[31] Farooq S, Hassan MM, Ruthven DM (1988). Heat effects in pressure swing adsorption systems. Chem. Eng. Sci..

[32] Tomomura M, Nogita S, Someya K (1987). Carbon dioxide removal from compressed air by pressure swing adsorption. Kagaku Kogaku Ronbunshu.

[33] Wilson SJ, Webley PA (2002). Cyclic steady-state axial temperature profiles in multilayer, bulk gas PSA—the case of oxygen VSA. Ind. Eng. Chem. Res..

[34] Sotomayor FJ, Lastoskie CM (2017). Predicting the breakthrough performance of gating adsorbents using osmotic framework-adsorbed solution theory. Langmuir.

[35] Yang S-I, Choi D-Y, Jang S-C, Kim S-H, Choi D-K (2008). Hydrogen separation by multi-bed pressure swing adsorption of synthesis gas. Adsorption.

[36] Maring BJ, Webley PA (2013). A new simplified pressure/vacuum swing adsorption model for rapid adsorbent screening for CO_2_ capture applications. Int. J. Greenh. Gas. Con.

[37] Yamazaki T, Takahashi Y, Yoshida D (2013). Adsorption of several gases on flexible metal organic framework [Cu(dhbc)_2_(4,4′-bpy)]·H_2_O. J. Colloid Interface Sci..

[38] Thommes M (2015). Physisorption of gases, with special reference to the evaluation of surface area and pore size distribution (IUPAC technical report). Pure Appl. Chem..

[39] Hefti M, Joss L, Bjelobrk Z, Mazzotti M (2016). On the potential of phase-change adsorbents for CO_2_ capture by temperature swing adsorption. Faraday Discuss..

[40] Saima WH, Mogi Y, Haraoka T (2013). Development of PSA system for the recovery of carbon dioxide and carbon monoxide from blast furnace gas in steel works. Energy Procedia.

[41] Tanaka H (2014). Adsorption-induced structural transition of ZIF-8: a combined experimental and simulation study. J. Phys. Chem. C..

[42] Kawaguchi S (2017). High-throughput powder diffraction measurement system consisting of multiple MYTHEN detectors at beamline BL02B2 of SPring-8. Rev. Sci. Instrum..

[43] Kawaguchi S (2020). Fast continuous measurement of synchrotron powder diffraction synchronized with controlling gas and vapour pressures at beamline BL02B2 of SPring-8. J. Synchrotron Rad..

[44] Delley B (1990). An all-electron numerical method for solving the local density functional for polyatomic molecules. J. Chem. Phys..

[45] Delley B (2000). From molecules to solids with the DMol^3^ approach. J. Chem. Phys..

[46] Rappé AK, Casewit CJ, Colwell KS, Goddard WA, Skiff WM (1992). UFF, a full periodic table force field for momechanics and molecular dynamics simulations. J. Am. Chem. Soc..

[47] Chen YF, Lee JY, Babarao R, Li J, Jiang JW (2010). A highly hydrophobic metal−organic framework Zn(BDC)(TED)_0.5_ for adsorption and separation of CH_3_OH/H_2_O and CO_2_/CH_4_: an integrated experimental and simulation study. J. Phys. Chem. C..

[48] Cracknell RF, Nicholson D (1996). Adsorption and selectivity of carbon dioxide with methane and nitrogen in slit-shaped carbonaceous micropores: simulation and experiment. Adsorption.

[49] Dubbeldam D, Calero S, Ellis DE, Snurr RQ (2016). RASPA: molecular simulation software for adsorption and diffusion in flexible nanoporous materials. Mol. Simul..

[50] Kresse G, Furthmüller J (1996). Efficiency of ab-initio total energy calculations for metals and semiconductors using a plane-wave basis set. Comput. Mater. Sci..

[51] Kresse G, Furthmüller J (1996). Efficient iterative schemes for ab initio total-energy calculations using a plane-wave basis set. Phys. Rev. B.

[52] Togo A, Tanaka I (2015). First principles phonon calculations in materials science. Scr. Mater..

[53] Blöchl PE (1994). Projector augmented-wave method. Phys. Rev. B.

[54] Numaguchi R, Tanaka H, Watanabe S, Miyahara MT (2013). Simulation study for adsorption-induced structural transition in stacked-layer porous coordination polymers: equilibrium and hysteretic adsorption behaviors. J. Chem. Phys..

