# Supplementary material for: High-throughput gas separation by flexible metal–organic frameworks with fast gating and thermal management capabilities
Source: Nat Commun. 2020 Aug 3;11:3867. doi: 10.1038/s41467-020-17625-3 (PMC7400644; doi:10.1038/s41467-020-17625-3)
Supplement: Supplementary file 1 — Supplementary Information [file 41467_2020_17625_MOESM1_ESM.pdf]

Supplementary Information

High-throughput gas separation by flexible metal–organic frameworks with fast gating and thermal management capabilities

Hiraide *et al.*

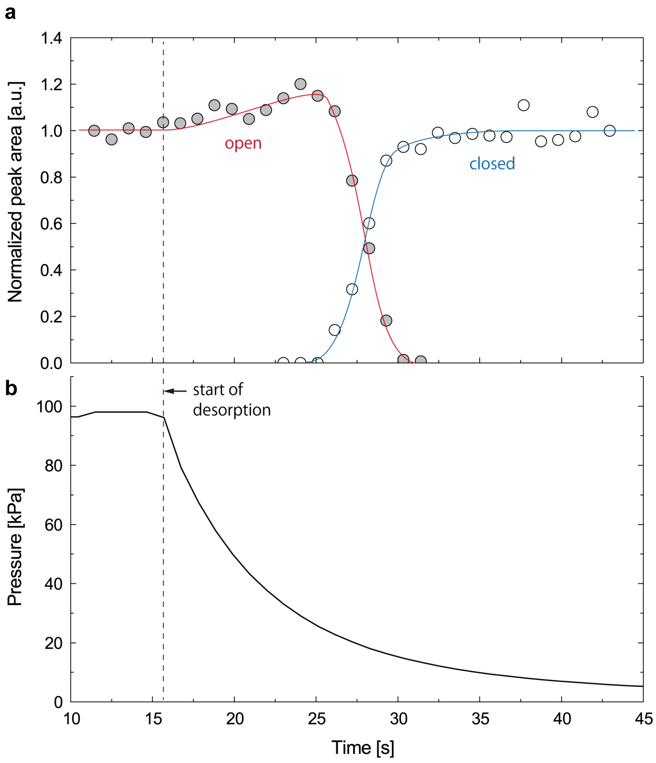

Supplementary Figure 1: Time-resolved *in situ* synchrotron XRPD. a Changes of normalized peak area obtained from the data measured by time-resolved *in situ* synchrotron XRPD for CO_2 desorption from ELM-11 at 273 K. The normalized peak areas of the 020 reflection at 10.2º for the closed phase and the 002 reflection at 6.1º for the open phase were evaluated by the nonlinear least-squares fitting of the pseudo-Voigt function. The CO_2 gas was depressed through a needle valve and the change of pressure is shown in **b**.

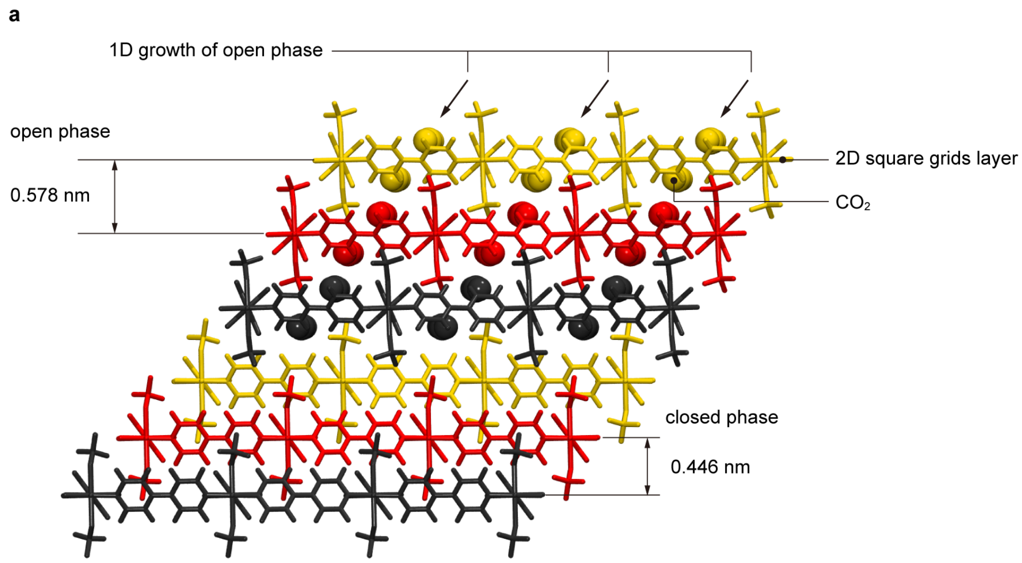

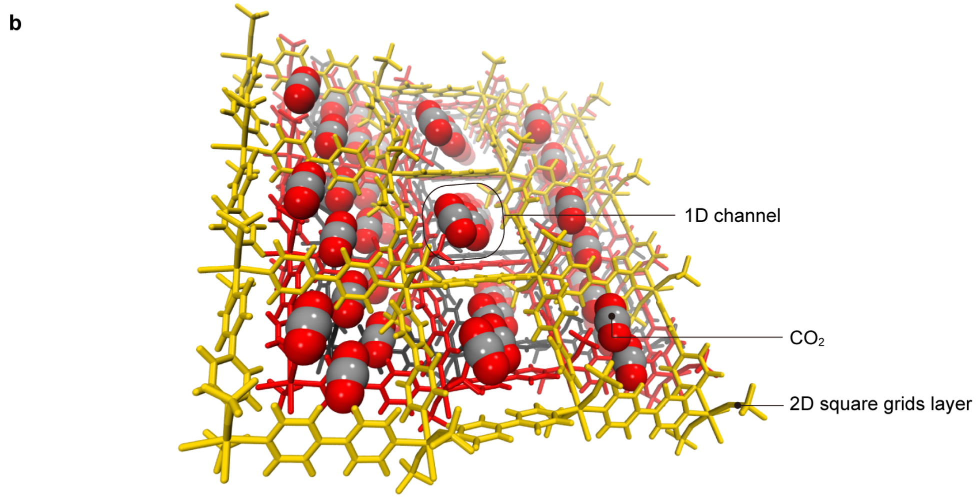

Supplementary Figure 2: Framework structure of ELM-11. a Schematic illustration of the 1D growth of open phase of ELM-11, **b** Snapshot of the open phase of ELM-11 showing the 1D channel.

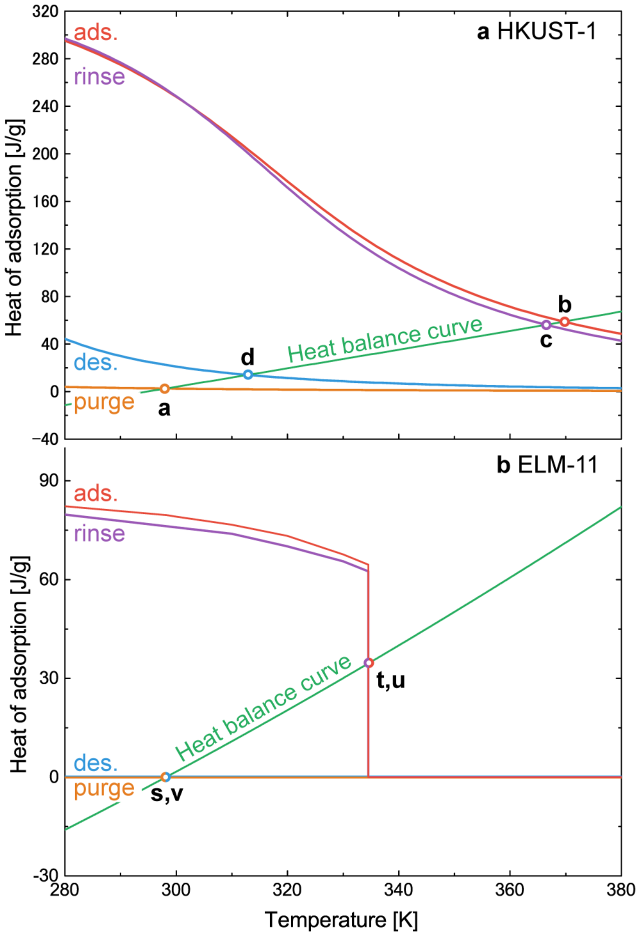

Supplementary Figure 3: Temperature dependence of the heat of adsorption for HKUST-1 and ELM-11. Red line: an equimolar CO_2/CH_4 gas mixture adsorption at 500 kPa, purple line: pure CO_2 adsorption at 250 kPa, blue line: pure CO_2 adsorption at 15 kPa, and yellow line: pure CH_4 adsorption at 15 kPa. The green line shows the heat required to change the temperature of the host framework obtained by integrating the specific heat with respect to temperature. The starting temperature was set to 298 K.

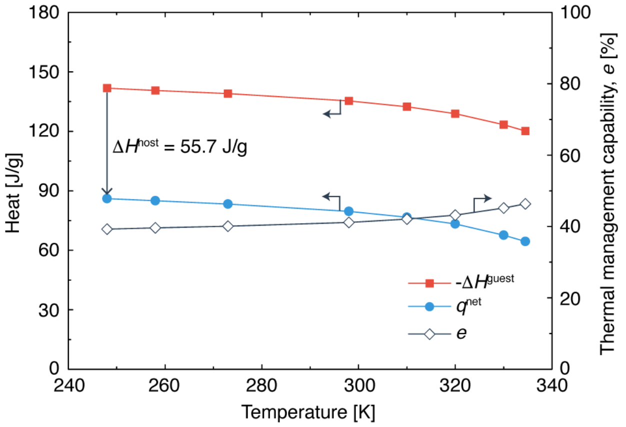

Supplementary Figure 4: Temperature dependence of the thermal management capability of ELM-11 during the adsorption process for an equimolar CO_2/CH_4 mixture gas at 500 kPa. $-\Delta H^{guest}$ is the exothermic heat, q^{net} the net heat, and e the thermal management capability. We assumed that the enthalpy change of host framework ΔH^{host} was equal to ΔU^{host} = 55.7 J/g.

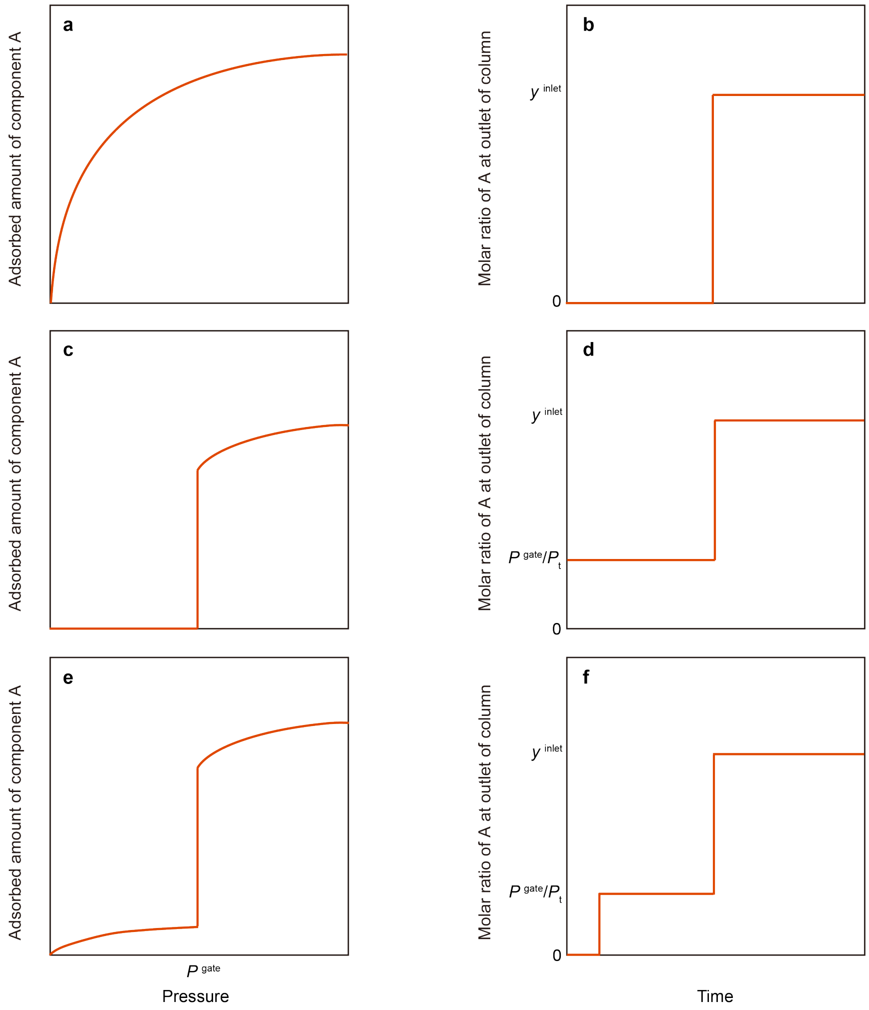

Supplementary Figure 5: Schematic illustrations of the relationship between the adsorption isotherm and the breakthrough curve. An adsorbent showing a Langmuir-type isotherm (**a**) provides a typical breakthrough curve (**b**), where y^{inlet} is the molar ratio of component **A** in the gas mixture at an inlet of the adsorption column. On the other hand, a flexible MOF showing a typical gate adsorption isotherm (**c**) gives breakthrough curve **d**. The non-zero concentration of component **A** (= $P^{\mathrm{gate}}/P_{\mathrm{t}}$, where P^{gate} is the gate adsorption pressure for pure **A** on the flexible MOF and P_{t} is the total pressure of the mixture gas) is due to the slipping-off phenomenon, which comes from the fact that the flexible MOF can no longer adsorb the component **A** when the partial pressure of component **A** in the gas mixture flowing in the adsorption column decreases below P^{gate}. The concentration of slipping-off component **A** is constant until the intrinsic breakthrough of component **A** occurs. A flexible MOF showing a type IV like isotherm (**e**) also exhibits the slipping-off phenomenon (breakthrough curve **f**), while there is a delay in the elusion of slipping-off component **A** because the amount adsorbed below the gate adsorption pressure is not zero. A simple mixture of an adsorbent showing isotherm **a** and a flexible MOF showing isotherm **b** provides isotherm **e**, and thus its breakthrough curve should be like breakthrough curve **f**.

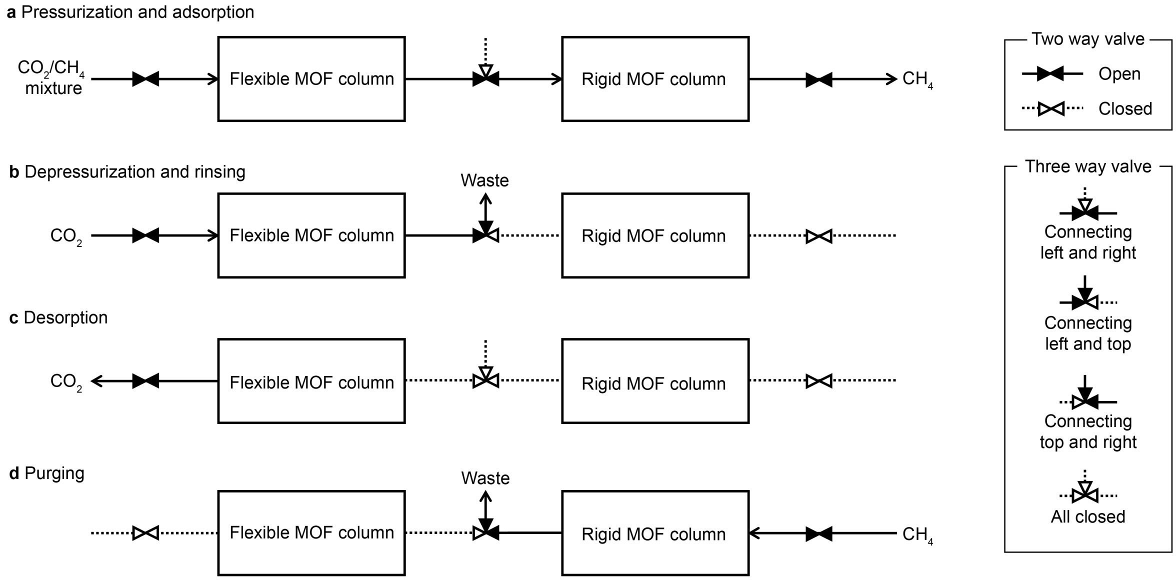

Supplementary Figure 6: Schematic illustration of sequential columns system with a three-way valve. The three-way valve enables us to perform different operations for the flexible-MOF column and the rigid-MOF column individually: **a** co-current pressurization and adsorption process for the two columns, **b** co-current depressurization and rinsing process only for the flexible-MOF column, **c** counter-current desorption process only for the flexible-MOF column, and **d** counter-current purging only for the rigid-MOF column. The efficiency of the combined columns system composed of ELM-11 and HKUST-1 can be further improved as follows. For example, the depressurization and rinsing process for the HKUST-1 column can be skipped because only a small amount of CO_2 is adsorbed in the column and it can be desorbed as a waste gas by the following purging process using pure CH_4. The depressurization from 500 to 250 kPa and rinsing process using pure CO_2 of 250 kPa is thus only required for the ELM-11 column. The advantage of these individual treatments is that only a small amount of CO_2 is needed for the rinsing process, which is because of the gate adsorption characteristic and large CO_2 selectivity of ELM-11. Then, the desorption process is only required for the ELM-11 column. Moreover, we can omit the purging process using pure CH_4 for the ELM-11 column because the small amount of effluent CO_2 from the ELM-11 column at the pressurization and adsorption process (the remained CO_2 on ELM-11 after the desorption process) can be adsorbed by the HKUST-1 column, which can suppress the consumption of the product CH_4.

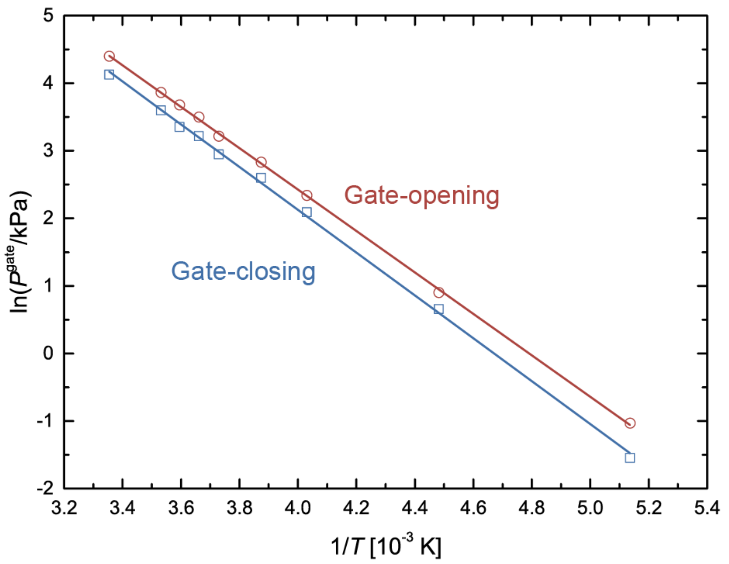

Supplementary Figure 7: lnP^{gate} vs. 1/T plot. Red circles: experimental gate-opening pressure of CO_2, blue squares: experimental gate-closing pressure of CO_2. Two lines are the results of the least-squares fitting to the experimental data.

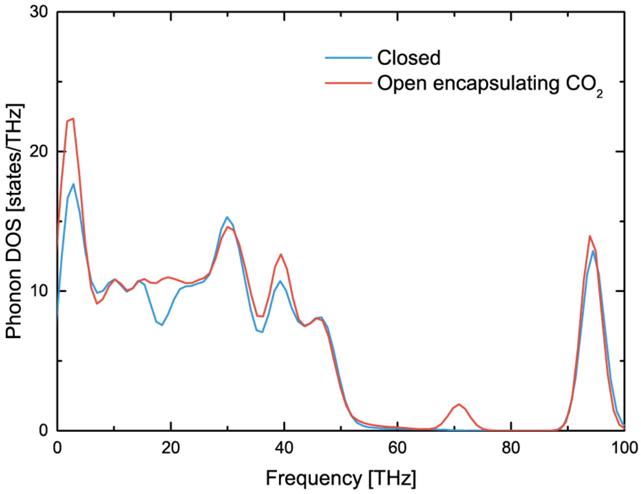

Supplementary Figure 8: Phonon density of states of ELM-11 in the closed and open states. The phonon density of states of the closed state is similar to that of the open state except for the peak at 70 THz coming from the C=O stretching vibration of adsorbed CO_2 molecules.

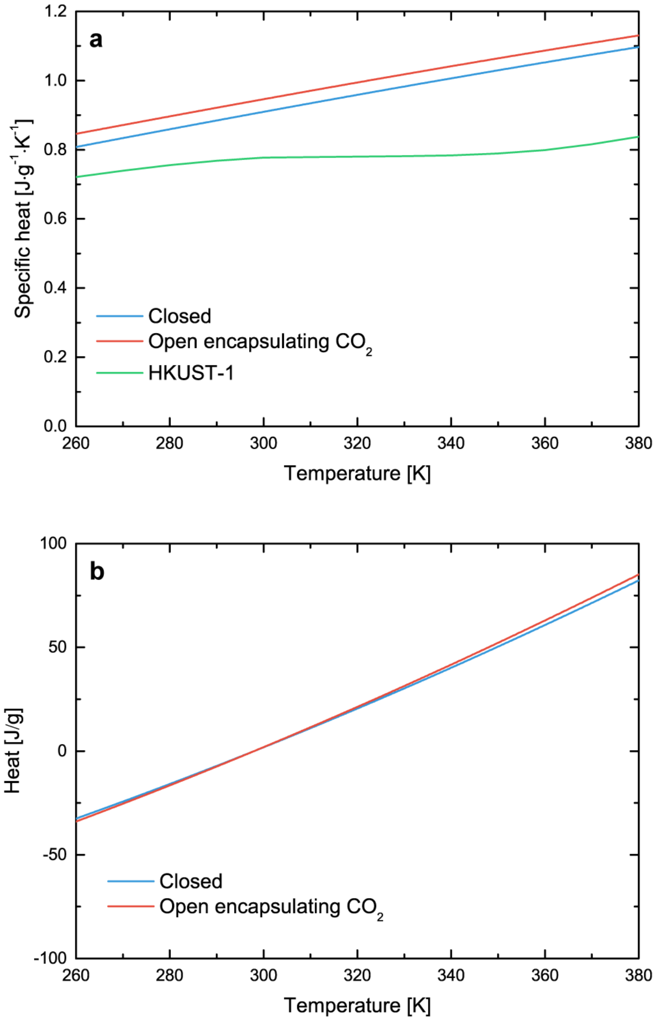

Supplementary Figure 9: Specific heat of ELM-11 and HKUST-1. a Temperature dependence of the specific heat of ELM-11 in the closed (blue) and open (red) states and HKUST-1 (green). The specific heat of the open structure is slightly different from that of the closed structure because of the existence of CO_2 molecules and the difference in the interlayer distance of the host framework. **b** The enthalpy change of ELM-11 in the closed (blue) and open (red) states. Since the difference between the two curves is quite small, we used the specific heat of the closed structure to compute the temperature change of ELM-11 (the green plane in Figure 5b) for the sake of simplicity.

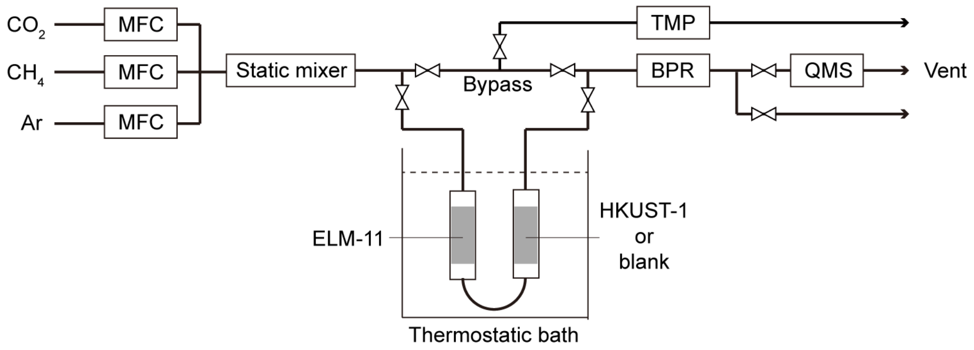

Supplementary Figure 10: Flowsheet of the breakthrough curve measurement. MFC: mass flow controller, TMP: turbo molecular pump, BPR: back-pressure regulator, and QMS: quadrupole mass spectrometer.

Supplementary Table 1: Parameters obtained from fitting of the KJMA equation to the fraction of the phase transformed versus time plots.

T [K]	P^{gate} [kPa]	P [kPa]	$P-P^{\text{gate}}$	n		k [$s^{-1/n}$]	
				open phase	closed phase	open phase	closed phase
273	31.7	40.8	9.09	1.31 ± 0.08	1.33 ± 0.1	0.10 ± 0.02	0.13 ± 0.03
264	21.7	41.0	19.4	1.30 ± 0.09	1.3 ± 0.2	0.23 ± 0.03	0.25 ± 0.08
256	15.1	40.8	25.8	1.2 ± 0.1	1.25 ± 0.07	0.33 ± 0.05	0.40 ± 0.03
241	7.15	40.8	33.6	1.2 ± 0.1	1.2 ± 0.1	0.40 ± 0.06	0.42 ± 0.06
227	3.26	7.74	4.48	1.26 ± 0.06	1.24 ± 0.09	0.036 ± 0.005	0.049 ± 0.006
		13.0	9.74	1.22 ± 0.09	1.2 ± 0.1	0.085 ± 0.02	0.097 ± 0.03
		16.9	13.6	1.24 ± 0.06	1.2 ± 0.1	0.16 ± 0.02	0.18 ± 0.04
		31.0	27.7	1.2 ± 0.1	1.22 ± 0.08	0.33 ± 0.04	0.40 ± 0.04
		40.9	37.6	1.2 ± 0.2	1.2 ± 0.2	0.5 ± 0.2	0.6 ± 0.1

Supplementary Table 2: Fractional coordinates, LJ parameters, and atomic charges of the open host framework of ELM-11.

Atom	x [-]	y [-]	z [-]	σ_{UFF} [nm]	$\alpha\varepsilon_{UFF}$ / k_B [K]	q [e]
Cu	0	0.19281	0.75	0.3114	1.863	0.621
B	0.19563	0.11984	0.64423	0.3638	67.061	0.788
F1	0.27073	0.06941	0.69054	0.2997	18.628	–0.390
F2	0.23654	0.19253	0.59436	0.2997	18.628	–0.365
F3	0.13256	0.19178	0.68463	0.2997	18.628	–0.426
F4	0.14045	0.02442	0.60743	0.2997	18.628	–0.433
N1	0.09943	0.19919	0.83834	0.3261	25.707	–0.400
N2	0	0.38035	0.75	0.3261	25.707	–0.446
N3	0	0.01305	0.75	0.3261	25.707	–0.412
C1	0.17252	0.27971	0.84012	0.3431	39.119	0.087
C2	0.23247	0.30018	0.90425	0.3431	39.119	–0.136
C3	0.21649	0.23863	0.96391	0.3431	39.119	0.061
C4	0.14311	0.15790	0.96174	0.3431	39.119	–0.112
C5	0.08398	0.13864	0.89771	0.3431	39.119	0.071
C6	0.04017	0.44108	0.69691	0.3431	39.119	0.104
C7	0.04084	0.56606	0.69588	0.3431	39.119	–0.058
C8	0	0.62901	0.75	0.3431	39.119	0.048
C9	0	0.76257	0.75	0.3431	39.119	–0.011
C10	0.08639	0.82586	0.77173	0.3431	39.119	–0.100
C11	0.08469	0.95217	0.77137	0.3431	39.119	0.101
H1	0.18536	0.32892	0.79222	0.2571	16.392	0.161
H2	0.12980	0.10800	1.00929	0.2571	16.392	0.185
H3	0.29137	0.36522	0.90578	0.2571	16.392	0.121
H4	0.02503	0.07370	0.89658	0.2571	16.392	0.159
H5	0.07159	0.39088	0.65549	0.2571	16.392	0.159
H6	0.07267	0.61354	0.65360	0.2571	16.392	0.185
H7	0.15060	1.00245	0.78805	0.2571	16.392	0.178
H8	0.15366	0.77845	0.78860	0.2571	16.392	0.158

Supplementary Table 3: The unit cell of the open host framework of ELM-11.

crystal system	monoclinic
space group	*C*2/*c* (No. 15)
a [nm]	1.37219
b [nm]	1.10542
c [nm]	1.87532
β [deg]	95.924

Supplementary Table 4: Interaction parameters for a CO_2 molecule.

site C			
	σ	[nm]	0.2789
	ε / k_B	[K]	29.66
	q	[e]	+0.576
site O			
	σ	[nm]	0.3011
	ε / k_B	[K]	82.96
	q	[e]	−0.288
C-O bond length		[nm]	0.118
O-C-O bond angle		[deg]	180

Supplementary Table 5: Interaction parameters for a CH_4 molecule.

σ	[nm]	0.381
ε / k_B	[K]	148.12

Supplementary Table 6: Parameters of the polynomial function for the specific heat of HKUST-1. $C[\mathrm{J\ g^{-1}\ K^{-1}}] = \sum_{i=0}^{5} a_i T^i$, unit of a_i: [J g^{-1} K$^{-(1+i)}$].

Parameters	80 K < T ≤ 300 K (Kloutse *et al.*)	300 K < T < 400 K
a_0	−0.426	448.0867
a_1	1.239×10^{-2}	−6.663567
a_2	-7.2796×10^{-5}	3.95955×10^{-2}
a_3	2.427×10^{-7}	-1.172852×10^{-4}
a_4	-3.105×10^{-10}	1.731346×10^{-7}
a_5	0	-1.018616×10^{-10}

Supplementary Table 7: Parameters of Virial-Langmuir model for CO_2 and CH_4 on HKUST-1 by Chowdhury *et al.*

Parameters		CO_2	CH_4
β_0	[mmol·g^{-1}·bar^{-1}]	5.64×10^{-4}	1.68×10^{-3}
β_1	[K]	2774	1931
b_0	[mmol^{-1}·g]	0.45	0.24
b_1	[mmol^{-1}·g·K]	−157.5	−59.3
c_0	[mmol^{-2}·g^{2}]	−0.069	−0.068
c_1	[mmol^{-2}·g^{2}·K]	21.8	17.2
η_0	[mmol^{-1}·g]	−8.29	−4.02
η_1	[mmol^{-1}·g·K]	7643	4635

Supplementary Note 1: Co-existence of the closed and open states of ELM-11.

Supplementary Figure 11 shows an adsorption isotherm of CO_2 on ELM-11 at 298 K and *in situ* XRPD patterns measured at the points designated by the numbers on the adsorption isotherm. Points 1 and 10 represent ELM-11 in the fully closed sand fully open states, respectively. The XRPD patterns at points 2–9 have both peaks attributed to the closed and peaks assigned to the open states, and the ratio of the two phases in the XRPD patterns at points 2–9 gradually changes with increasing CO_2 pressure. This result indicates that the closed and open states of ELM-11 co-exist during the gate-opening. This fact supports the assumption that only a part of ELM-11 can undergo gate adsorption during the adiabatic adsorption process to satisfy the heat balance of the system.

Supplementary Figure 12 schematically illustrates the co-existence of the closed and open states of ELM-11 under the adiabatic adsorption process. State A: all of the ELM-11 crystals are in the closed state and at the same temperature T_0 just after the adiabatic purging process. State B: by feeding an equimolar CO_2/CH_4 gas mixture, a portion of ELM-11 (54% when T_0 = 298 K) adsorbs CO_2 and releases the heat of adsorption. State C: The heat released by the gate adsorption increases the temperature of ELM-11 crystals in both closed and open states, and then the system achieves thermal equilibrium at T^{gate} (335 K for an equimolar CO_2/CH_4 gas mixture at 500 kPa).

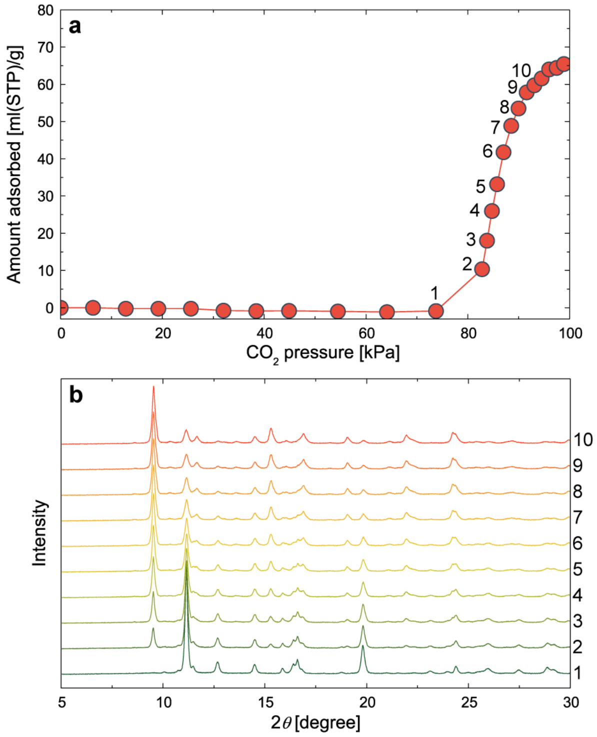

Supplementary Figure 11: *In situ* XRPD measurements. a Adsorption isotherms of CO_2 on ELM-11 at 298 K. **b** *In situ* XRPD patterns measured at the points designated by the numbers in **a**.

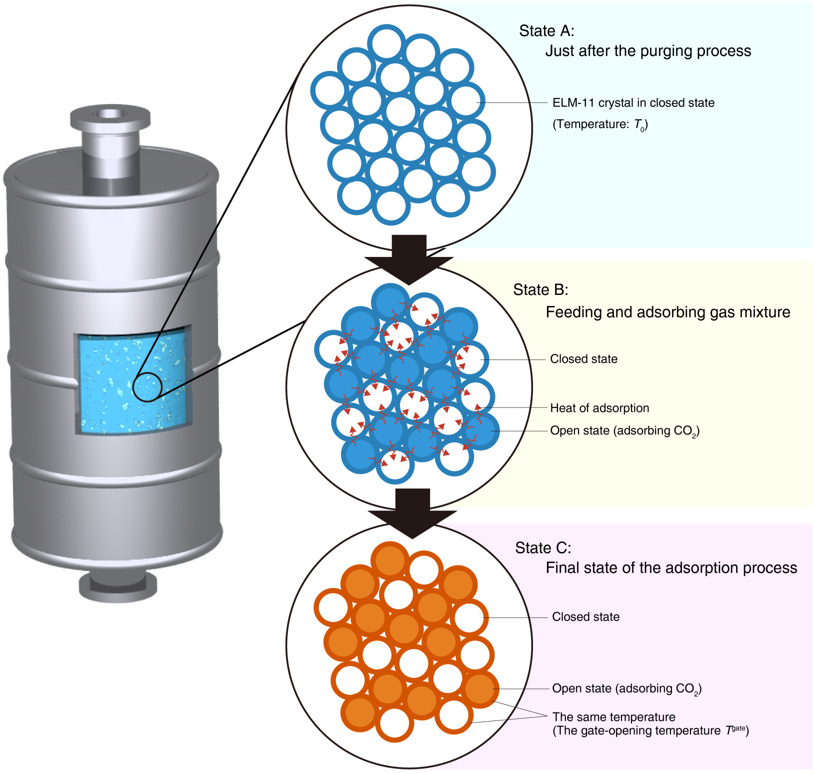

Supplementary Figure 12: Schematic illustration of the co-existence of the closed and open states of ELM-11 under the adiabatic adsorption process.

Supplementary Methods

Material balance calculations for the conventional system and our proposed system

In this section, we designate the conventional system as System **A** and our proposed system as System **B**.

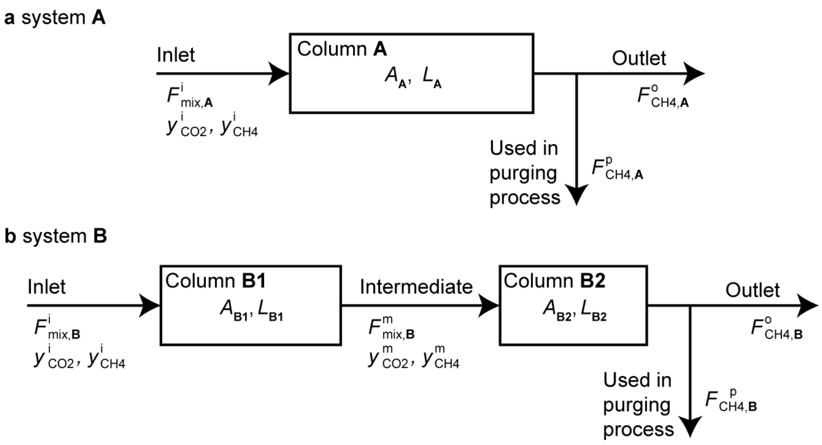

Supplementary Figure 13. Schematic diagram of system A and system B.

System A

Supplementary Figure 13a shows a schematic diagram of system **A**. $F_{j,\mathrm{A}}$ and y_j are the molar flow rate and the fraction of component j (= CO_2, CH_4, and mix, where mix denotes a CO_2/CH_4 gas mixture). The superscripts for $F_{j,\mathrm{A}}$ and y_j, "i," "p," and "o," stand for inlet, purge, and outlet streams, respectively. The cross-sectional area of the column is A_A and the length of the column is L_A. Material balance equations for CO_2 and CH_4 associated with column **A** filled with a rigid MOF when the total inflow becomes equal to the maximum adsorption capacity of the column are given by:

$$y^{\mathrm{i}}_{\mathrm{CO2}}F^{\mathrm{i}}_{\mathrm{mix,\mathbf{A}}} = \frac{L_{\mathbf{A}}A_{\mathbf{A}}(1-\varepsilon_{\mathbf{A}})}{\theta_{\mathbf{A}}}N^{\mathrm{ads}}_{\mathrm{CO2,\mathbf{A}}}, \tag{S1}$$

$$y^{\mathrm{i}}_{\mathrm{CH4}}F^{\mathrm{i}}_{\mathrm{mix,\mathbf{A}}} = \frac{L_{\mathbf{A}}A_{\mathbf{A}}(1-\varepsilon_{\mathbf{A}})}{\theta_{\mathbf{A}}}N^{\mathrm{ads}}_{\mathrm{CH4,\mathbf{A}}} + F^{\mathrm{P}}_{\mathrm{CH4,\mathbf{A}}} + F^{\mathrm{o}}_{\mathrm{CH4,\mathbf{A}}}, \tag{S2}$$

where, $\varepsilon_{\mathbf{A}}$ is the overall void fraction of column **A**, $\theta_{\mathbf{A}}$ is the operating time for the adsorption process for system **A**, $N^{\mathrm{ads}}_{j,\mathbf{A}}$ is the effective adsorption amount of component j for the rigid MOF on the volumetric basis, which depends on the adiabatic operation conditions. The CH_4 flow required for the purging process, $F^{\mathrm{P}}_{\mathrm{CH4,\mathbf{A}}}$, would be proportional to the CO_2 amount remained in the rigid MOF after the desorption process:

$$F^{\mathrm{P}}_{\mathrm{CH4,\mathbf{A}}} = \alpha_{\mathbf{A}}\frac{L_{\mathbf{A}}A_{\mathbf{A}}(1-\varepsilon_{\mathbf{A}})}{\theta_{\mathbf{A}}}N^{\mathrm{des}}_{\mathrm{CO2,\mathbf{A}}}, \tag{S3}$$

where, $\alpha_{\mathbf{A}}$ is a proportionality factor representing the effectiveness of purging. According to the definition of the separation characteristics for PVSA process, the adsorption amount of CH_4 under the adiabatic adsorption process, $N^{\mathrm{ads}}_{\mathrm{CH4,\mathbf{A}}}$, is a function of the CO_2 selectivity, $S^{\mathrm{ads}}_{\mathbf{A}}$, which is written as:

$$N^{\mathrm{ads}}_{\mathrm{CH4,\mathbf{A}}} = N^{\mathrm{ads}}_{\mathrm{CO2,\mathbf{A}}}\frac{y^{\mathrm{i}}_{\mathrm{CH4}}/y^{\mathrm{i}}_{\mathrm{CO2}}}{S^{\mathrm{ads}}_{\mathbf{A}}}. \tag{S4}$$

Then, the adsorption amount of CO_2 under the adiabatic desorption process, $N^{\mathrm{des}}_{\mathrm{CO2},\mathbf{A}}$, is obtained from the regenerability, $R_{\mathbf{A}}$, and $N^{\mathrm{ads}}_{\mathrm{CO2},\mathbf{A}}$ as:

$$N^{\mathrm{des}}_{\mathrm{CO2},\mathbf{A}} = N^{\mathrm{ads}}_{\mathrm{CO2},\mathbf{A}}(1 - R_{\mathbf{A}}). \tag{S5}$$

The length of column **A** and the molar flow rate of the product CH_4 can be obtained by simultaneously solving Eqns. S1–S5:

$$L_{\mathbf{A}} = \frac{\theta_{\mathbf{A}}}{A_{\mathbf{A}}(1-\varepsilon_{\mathbf{A}})} \frac{y^{\mathrm{i}}_{\mathrm{CO2}}}{N^{\mathrm{ads}}_{\mathrm{CO2},\mathbf{A}}} F^{\mathrm{i}}_{\mathrm{mix},\mathbf{A}}, \tag{S6}$$

$$\begin{aligned} F^{\mathrm{o}}_{\mathrm{CH4},\mathbf{A}} &= y^{\mathrm{i}}_{\mathrm{CH4}} F^{\mathrm{i}}_{\mathrm{mix},\mathbf{A}} - \frac{L_{\mathbf{A}} A_{\mathbf{A}}(1-\varepsilon_{\mathbf{A}})}{\theta_{\mathbf{A}}} N^{\mathrm{ads}}_{\mathrm{CO2},\mathbf{A}} \frac{y^{\mathrm{i}}_{\mathrm{CH4}}/y^{\mathrm{i}}_{\mathrm{CO2}}}{S^{\mathrm{ads}}_{\mathbf{A}}} - \alpha_{\mathbf{A}} \frac{L_{\mathbf{A}} A_{\mathbf{A}}(1-\varepsilon_{\mathbf{A}})}{\theta_{\mathbf{A}}} N^{\mathrm{ads}}_{\mathrm{CO2},\mathbf{A}}(1-R_{\mathbf{A}}) \\ &= y^{\mathrm{i}}_{\mathrm{CH4}} F^{\mathrm{i}}_{\mathrm{mix},\mathbf{A}} - \frac{L_{\mathbf{A}} A_{\mathbf{A}}(1-\varepsilon_{\mathbf{A}})}{\theta_{\mathbf{A}}} N^{\mathrm{ads}}_{\mathrm{CO2},\mathbf{A}} \left\{ \frac{y^{\mathrm{i}}_{\mathrm{CH4}}/y^{\mathrm{i}}_{\mathrm{CO2}}}{S^{\mathrm{ads}}_{\mathbf{A}}} + \alpha_{\mathbf{A}}(1-R_{\mathbf{A}}) \right\} \\ &= \left[y^{\mathrm{i}}_{\mathrm{CH4}} - y^{\mathrm{i}}_{\mathrm{CO2}} \left\{ \frac{y^{\mathrm{i}}_{\mathrm{CH4}}/y^{\mathrm{i}}_{\mathrm{CO2}}}{S^{\mathrm{ads}}_{\mathbf{A}}} + \alpha_{\mathbf{A}}(1-R_{\mathbf{A}}) \right\} \right] F^{\mathrm{i}}_{\mathrm{mix},\mathbf{A}} \equiv x_{\mathbf{A}} F^{\mathrm{i}}_{\mathrm{mix},\mathbf{A}}. \end{aligned} \tag{S7}$$

System B

(i) Case I

Supplementary Figure 13b shows a schematic diagram of system **B**. $F_{j,\mathbf{B}}$ and y_j are the molar flow rate and the fraction of component j (= CO_2, CH_4, and mix). The superscripts for $F_{j,\mathbf{B}}$ and y_j, "i," "p," "o," and "m" stand for the streams at inlet, purge, outlet, and intermediate between columns **B1** and **B2**, respectively. The cross-sectional areas of columns **B1** and **B2** are $A_{\mathbf{B1}}$ and $A_{\mathbf{B2}}$, and the lengths of them are $L_{\mathbf{B1}}$ and $L_{\mathbf{B2}}$. Material balance equations for CO_2 and CH_4 associated with column **B1** filled with a flexible MOF before the intrinsic breakthrough of CO_2 are given by:

$$y^{\mathrm{i}}_{\mathrm{CO2}} F^{\mathrm{i}}_{\mathrm{mix},\mathbf{B}} = \frac{L_{\mathbf{B1}} A_{\mathbf{B1}}(1-\varepsilon_{\mathbf{B1}})}{\theta_{\mathbf{B}}} N^{\mathrm{ads}}_{\mathrm{CO2},\mathbf{B1}} + y^{\mathrm{m}}_{\mathrm{CO2}} F^{\mathrm{m}}_{\mathrm{mix},\mathbf{B}}, \tag{S8}$$

$$y^{\mathrm{i}}_{\mathrm{CH4}} F^{\mathrm{i}}_{\mathrm{mix},\mathbf{B}} = \frac{L_{\mathbf{B1}} A_{\mathbf{B1}}(1-\varepsilon_{\mathbf{B1}})}{\theta_{\mathbf{B}}} N^{\mathrm{ads}}_{\mathrm{CH4},\mathbf{B1}} + y^{\mathrm{m}}_{\mathrm{CH4}} F^{\mathrm{m}}_{\mathrm{mix},\mathbf{B}}, \tag{S9}$$

where, $\varepsilon_{\mathbf{B1}}$ is the overall void fraction of column **B1**, $\theta_{\mathbf{B}}$ is the operating time for the adsorption process for system **B**, $N^{\mathrm{ads}}_{j,\mathbf{B1}}$ is the effective adsorption amount of component j for the flexible MOF on the volumetric basis, and the CO_2 fraction of intermediate flow, $y^{\mathrm{m}}_{\mathrm{CO2}}$, is governed by the gate adsorption pressure of the flexible MOF for pure CO_2, P^{gate}, and the total pressure in the adsorption column, P_{t}, ($y^{\mathrm{m}}_{\mathrm{CO2}} = P^{\mathrm{gate}}/P_{\mathrm{t}}$). The $N^{\mathrm{ads}}_{\mathrm{CH4},\mathbf{B1}}$ value is calculated from the CO_2 selectivity of the flexible MOF, $S^{\mathrm{ads}}_{\mathbf{B1}}$, and $N^{\mathrm{ads}}_{\mathrm{CO2},\mathbf{B1}}$ with the same relation as Eqn. S4.

The length of column **B1** and the molar flow rate of the intermediate stream can be obtained by simultaneously solving Eqns. S4, S8 and S9:

$$L_{\mathbf{B1}} = \frac{\theta_{\mathbf{B}}}{A_{\mathbf{B1}}(1-\varepsilon_{\mathbf{B1}})} \frac{y^{\mathrm{i}}_{\mathrm{CO2}}/y^{\mathrm{m}}_{\mathrm{CO2}} - y^{\mathrm{i}}_{\mathrm{CH4}}/y^{\mathrm{m}}_{\mathrm{CH4}}}{N^{\mathrm{ads}}_{\mathrm{CO2},\mathbf{B1}} \left(1/y^{\mathrm{m}}_{\mathrm{CO2}} - 1/y^{\mathrm{m}}_{\mathrm{CH4}} \frac{y^{\mathrm{i}}_{\mathrm{CH4}}/y^{\mathrm{i}}_{\mathrm{CO2}}}{S^{\mathrm{ads}}_{\mathbf{B1}}} \right)} F^{\mathrm{i}}_{\mathrm{mix},\mathbf{B}}, \tag{S10}$$

$$F_{\mathrm{mix,\mathbf{B}}}^{\mathrm{m}} = \frac{y_{\mathrm{CH4}}^{\mathrm{i}} - y_{\mathrm{CO2}}^{\mathrm{i}} \frac{y_{\mathrm{CH4}}^{\mathrm{i}}/y_{\mathrm{CO2}}^{\mathrm{i}}}{S_{\mathbf{B1}}^{\mathrm{ads}}}}{y_{\mathrm{CH4}}^{\mathrm{m}} - y_{\mathrm{CO2}}^{\mathrm{m}} \frac{y_{\mathrm{CH4}}^{\mathrm{i}}/y_{\mathrm{CO2}}^{\mathrm{i}}}{S_{\mathbf{B1}}^{\mathrm{ads}}}} F_{\mathrm{mix,\mathbf{B}}}^{\mathrm{i}}. \tag{S11}$$

Material balance equations for CO_2 and CH_4 associated with column **B2**, which is filled with a rigid MOF, can be written as:

$$y_{\mathrm{CO2}}^{\mathrm{m}} F_{\mathrm{mix,\mathbf{B}}}^{\mathrm{m}} = \frac{L_{\mathbf{B2}} A_{\mathbf{B2}} (1 - \varepsilon_{\mathbf{B2}})}{\theta_{\mathbf{B}}} N_{\mathrm{CO2,\mathbf{B2}}}^{\mathrm{ads}}, \tag{S12}$$

$$y_{\mathrm{CH4}}^{\mathrm{m}} F_{\mathrm{mix,\mathbf{B}}}^{\mathrm{m}} = \frac{L_{\mathbf{B2}} A_{\mathbf{B2}} (1 - \varepsilon_{\mathbf{B2}})}{\theta_{\mathbf{B}}} N_{\mathrm{CH4,\mathbf{B2}}}^{\mathrm{ads}} + F_{\mathrm{CH4,\mathbf{B}}}^{\mathrm{p}} + F_{\mathrm{CH4,\mathbf{B}}}^{\mathrm{o}}, \tag{S13}$$

where, $\varepsilon_{\mathbf{B2}}$ is the overall void fraction of column **B2**, $N_{j,\mathbf{B2}}^{\mathrm{ads}}$ is the effective adsorption amount of component j for the rigid MOF on the volumetric basis. The $N_{\mathrm{CH4,\mathbf{B2}}}^{\mathrm{ads}}$ value is calculated from the CO_2 selectivity of the rigid MOF, $S_{\mathbf{B2}}^{\mathrm{ads}}$, and $N_{\mathrm{CO2,\mathbf{B2}}}^{\mathrm{ads}}$ with the same relation as Eqn. S4. The stream for the purging process, $F_{\mathrm{CH4,\mathbf{B}}}^{\mathrm{p}}$, consists of two terms:

$$F_{\mathrm{CH4,\mathbf{B}}}^{\mathrm{p}} = \alpha_{\mathbf{B1}} \frac{L_{\mathbf{B1}} A_{\mathbf{B1}} (1 - \varepsilon_{\mathbf{B1}})}{\theta_{\mathbf{B}}} N_{\mathrm{CO2,\mathbf{B1}}}^{\mathrm{des}} + \alpha_{\mathbf{B2}} \frac{L_{\mathbf{B2}} A_{\mathbf{B2}} (1 - \varepsilon_{\mathbf{B2}})}{\theta_{\mathbf{B}}} N_{\mathrm{CO2,\mathbf{B2}}}^{\mathrm{des}}. \tag{S14}$$

where, $\alpha_{\mathbf{B1}}$ and $\alpha_{\mathbf{B2}}$ are proportionality factors representing the effectiveness of purging. The first term on the right-hand side of Eqn. S14 is attributed to the contribution from column **B1** and the second term is attributed to that from column **B2**. Note that the first term is zero when all the guest molecules on the flexible MOF is evacuated during the desorption process, which is the characteristic of flexible MOFs like ELM-11. The $N_{\mathrm{CO2,\mathbf{B2}}}^{\mathrm{des}}$ value on the second term can be calculated by the regenerability of the rigid MOF, $R_{\mathbf{B2}}$, and $N_{\mathrm{CO2,\mathbf{B2}}}^{\mathrm{ads}}$ with the same relation as Eqn. S5.

The length of column **B2** and the molar flow rate of the product CH_4 can be obtained as:

$$\begin{aligned} L_{\mathbf{B2}} &= \frac{\theta_{\mathbf{B}}}{A_{\mathbf{B2}} (1 - \varepsilon_{\mathbf{B2}})} \frac{y_{\mathrm{CO2}}^{\mathrm{m}}}{N_{\mathrm{CO2,\mathbf{B2}}}^{\mathrm{ads}}} F_{\mathrm{mix,\mathbf{B}}}^{\mathrm{m}} \\ &= \frac{\theta_{\mathbf{B}}}{A_{\mathbf{B2}} (1 - \varepsilon_{\mathbf{B2}})} \frac{y_{\mathrm{CO2}}^{\mathrm{m}}}{N_{\mathrm{CO2,\mathbf{B2}}}^{\mathrm{ads}}} \frac{y_{\mathrm{CH4}}^{\mathrm{i}} - y_{\mathrm{CO2}}^{\mathrm{i}} \frac{y_{\mathrm{CH4}}^{\mathrm{i}}/y_{\mathrm{CO2}}^{\mathrm{i}}}{S_{\mathbf{B1}}^{\mathrm{ads}}}}{y_{\mathrm{CH4}}^{\mathrm{m}} - y_{\mathrm{CO2}}^{\mathrm{m}} \frac{y_{\mathrm{CH4}}^{\mathrm{i}}/y_{\mathrm{CO2}}^{\mathrm{i}}}{S_{\mathbf{B1}}^{\mathrm{ads}}}} F_{\mathrm{mix,\mathbf{B}}}^{\mathrm{i}}, \end{aligned} \tag{S15}$$

$$\begin{aligned} F_{\mathrm{CH4,\mathbf{B}}}^{\mathrm{o}} &= y_{\mathrm{CH4}}^{\mathrm{m}} F_{\mathrm{mix,\mathbf{B}}}^{\mathrm{m}} - \frac{L_{\mathbf{B2}} A_{\mathbf{B2}} (1 - \varepsilon_{\mathbf{B2}})}{\theta_{\mathbf{B}}} N_{\mathrm{CO2,\mathbf{B2}}}^{\mathrm{ads}} \left\{ \frac{y_{\mathrm{CH4}}^{\mathrm{m}}/y_{\mathrm{CO2}}^{\mathrm{m}}}{S_{\mathbf{B2}}^{\mathrm{ads}}} + \alpha_{\mathbf{B2}} (1 - R_{\mathbf{B2}}) \right\} \\ &\quad - \alpha_{\mathbf{B1}} (1 - R_{\mathbf{B1}}) \frac{L_{\mathbf{B1}} A_{\mathbf{B1}} (1 - \varepsilon_{\mathbf{B1}})}{\theta_{\mathbf{B}}} N_{\mathrm{CO2,\mathbf{B1}}}^{\mathrm{ads}} \\ &= \left[y_{\mathrm{CH4}}^{\mathrm{m}} - y_{\mathrm{CO2}}^{\mathrm{m}} \left\{ \frac{y_{\mathrm{CH4}}^{\mathrm{m}}/y_{\mathrm{CO2}}^{\mathrm{m}}}{S_{\mathbf{B2}}^{\mathrm{ads}}} + \alpha_{\mathbf{B2}} (1 - R_{\mathbf{B2}}) \right\} \right] F_{\mathrm{mix,\mathbf{B}}}^{\mathrm{m}} \\ &\quad - \alpha_{\mathbf{B1}} (1 - R_{\mathbf{B1}}) \frac{y_{\mathrm{CO2}}^{\mathrm{i}}/y_{\mathrm{CO2}}^{\mathrm{m}} - y_{\mathrm{CH4}}^{\mathrm{i}}/y_{\mathrm{CH4}}^{\mathrm{m}}}{1/y_{\mathrm{CO2}}^{\mathrm{m}} - 1/y_{\mathrm{CH4}}^{\mathrm{m}} \frac{y_{\mathrm{CH4}}^{\mathrm{i}}/y_{\mathrm{CO2}}^{\mathrm{i}}}{S_{\mathbf{B1}}^{\mathrm{ads}}}} F_{\mathrm{mix,\mathbf{B}}}^{\mathrm{i}} \end{aligned}$$

$$\equiv x_{\mathbf{B}} F_{\text{mix},\mathbf{B}}^{\text{i}}, \tag{S16}$$

where

$$x_{\mathbf{B}} = \left[y_{\text{CH4}}^{\text{m}} - y_{\text{CO2}}^{\text{m}} \left\{ \frac{y_{\text{CH4}}^{\text{m}}/y_{\text{CO2}}^{\text{m}}}{S_{\mathbf{B2}}^{\text{ads}}} + \alpha_{\mathbf{B2}}(1 - R_{\mathbf{B2}}) \right\} \right] \frac{y_{\text{CH4}}^{\text{i}} - y_{\text{CO2}}^{\text{i}} \dfrac{y_{\text{CH4}}^{\text{i}}/y_{\text{CO2}}^{\text{i}}}{S_{\mathbf{B1}}^{\text{ads}}}}{y_{\text{CH4}}^{\text{m}} - y_{\text{CO2}}^{\text{m}} \dfrac{y_{\text{CH4}}^{\text{i}}/y_{\text{CO2}}^{\text{i}}}{S_{\mathbf{B1}}^{\text{ads}}}}$$

$$- \alpha_{\mathbf{B1}}(1 - R_{\mathbf{B1}}) \frac{y_{\text{CO2}}^{\text{i}}/y_{\text{CO2}}^{\text{m}} - y_{\text{CH4}}^{\text{i}}/y_{\text{CH4}}^{\text{m}}}{1/y_{\text{CO2}}^{\text{m}} - 1/y_{\text{CH4}}^{\text{m}} \dfrac{y_{\text{CH4}}^{\text{i}}/y_{\text{CO2}}^{\text{i}}}{S_{\mathbf{B1}}^{\text{ads}}}}. \tag{S17}$$

(ii) Case II

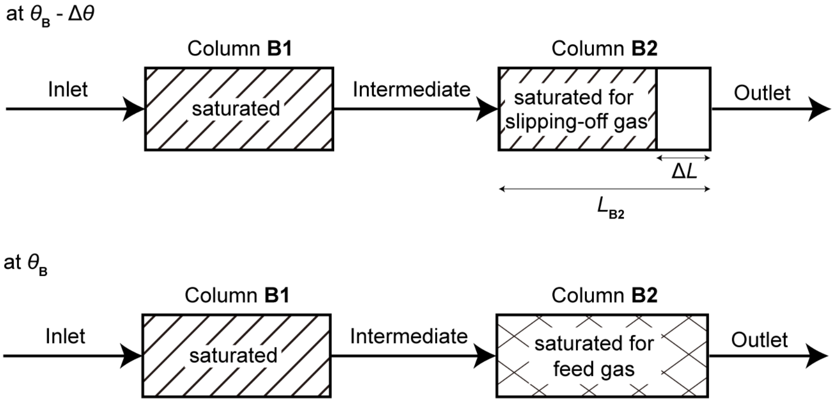

Supplementary Figure 14: Schematic diagram of the movement of the saturated zone in Case II.

In Case II, the length of column **B2** is defined such that the first breakpoint related to the slipping-off gas just merges with the second breakpoint associated with the feed gas. Namely, column **B2** is designed so that it breaks at the operation time $\theta_{\mathbf{B}}$ and column **B1** is designed so that it breaks earlier time than $\theta_{\mathbf{B}}$. As shown in Supplementary Figure 14, we consider that column **B1** breaks at $\theta_{\mathbf{B}} - \Delta\theta$, and ΔL of unadsorbed zone remains in column **B2** at that time. $\Delta\theta$ is derived from the following equations relating to the velocity of the mass-transfer zone:

$$L_{\mathbf{B2}} = \frac{y_{\text{CO2}}^{\text{m}} F_{\text{mix},\mathbf{B}}^{\text{m}}}{N_{\text{CO2},\mathbf{B2}}^{\text{ads}}(\text{slipping off}) A_{\mathbf{B2}}(1 - \varepsilon_{\mathbf{B2}})} \theta_{\mathbf{B}} \equiv r_{\text{gate2zero}} \theta_{\mathbf{B}}, \tag{S18}$$

$$L_{\mathbf{B2}} = \frac{y_{\text{CO2}}^{\text{i}} F_{\text{mix},\mathbf{B}}^{\text{i}} - y_{\text{CO2}}^{\text{m}} F_{\text{mix},\mathbf{B}}^{\text{m}}}{(N_{\text{CO2},\mathbf{B2}}^{\text{ads}}(\text{feed}) - N_{\text{CO2},\mathbf{B2}}^{\text{ads}}(\text{slipping off})) A_{\mathbf{B2}}(1 - \varepsilon_{\mathbf{B2}})} \Delta\theta \equiv r_{\text{feed2gate}} \Delta\theta, \tag{S19}$$

Where, $N_{\text{CO2},\mathbf{B2}}^{\text{ads}}(\text{slipping off})$ and $N_{\text{CO2},\mathbf{B2}}^{\text{ads}}(\text{feed})$ are saturated adsorption loadings for the slipping-off CO_2 and feed, respectively. Note that $r_{\text{gate2zero}}$ [m/s] is the velocity of the mass-transfer zone to reduce CO_2 fraction from $P^{\text{gate}}/P_{\text{t}}$ to zero and $r_{\text{feed2gate}}$ is that to reduce CO_2 fraction from y_{Feed} to $P^{\text{gate}}/P_{\text{t}}$ (see Figure 7). If the system satisfies $r_{\text{feed2gate}} > r_{\text{gate2zero}}$, the time lag between the first and second breakpoints will be zero by installing a little larger amount of conventional adsorbent in column **B2**. $\Delta\theta$ is obtained by

simultaneously solving Eqns. S21 and S22:

$$\Delta\theta = \frac{N^{\text{ads}}_{\text{CO2},\mathbf{B2}}(\text{feed}) - N^{\text{ads}}_{\text{CO2},\mathbf{B2}}(\text{slipping off})}{N^{\text{ads}}_{\text{CO2},\mathbf{B2}}(\text{slipping off})} \frac{y^{\text{m}}_{\text{CO2}} F^{\text{m}}_{\text{mix},\mathbf{B}}}{y^{\text{i}}_{\text{CO2}} F^{\text{i}}_{\text{mix},\mathbf{B}} - y^{\text{m}}_{\text{CO2}} F^{\text{m}}_{\text{mix},\mathbf{B}}} \theta_{\mathbf{B}}$$

$$= \frac{N^{\text{ads}}_{\text{CO2},\mathbf{B2}}(\text{feed}) - N^{\text{ads}}_{\text{CO2},\mathbf{B2}}(\text{slipping off})}{N^{\text{ads}}_{\text{CO2},\mathbf{B2}}(\text{slipping off})} \frac{y^{\text{m}}_{\text{CO2}} \dfrac{y^{\text{i}}_{\text{CH4}} - y^{\text{i}}_{\text{CO2}} \dfrac{y^{\text{i}}_{\text{CH4}}/y^{\text{i}}_{\text{CO2}}}{S^{\text{ads}}_{\mathbf{B1}}}}{y^{\text{m}}_{\text{CH4}} - y^{\text{m}}_{\text{CO2}} \dfrac{y^{\text{i}}_{\text{CH4}}/y^{\text{i}}_{\text{CO2}}}{S^{\text{ads}}_{\mathbf{B1}}}}}{y^{\text{i}}_{\text{CO2}} - y^{\text{m}}_{\text{CO2}} \dfrac{y^{\text{i}}_{\text{CH4}} - y^{\text{i}}_{\text{CO2}} \dfrac{y^{\text{i}}_{\text{CH4}}/y^{\text{i}}_{\text{CO2}}}{S^{\text{ads}}_{\mathbf{B1}}}}{y^{\text{m}}_{\text{CH4}} - y^{\text{m}}_{\text{CO2}} \dfrac{y^{\text{i}}_{\text{CH4}}/y^{\text{i}}_{\text{CO2}}}{S^{\text{ads}}_{\mathbf{B1}}}}} \theta_{\mathbf{B}}. \quad \text{(S20)}$$

The length of column **B1** is obtained by changing $\theta_{\mathbf{B}}$ of Eqn. S10 to $\theta_{\mathbf{B}} - \Delta\theta$:

$$L_{\mathbf{B1}} = \frac{\theta_{\mathbf{B}} - \Delta\theta}{A_{\mathbf{B1}}(1 - \varepsilon_{\mathbf{B1}})} \frac{y^{\text{i}}_{\text{CO2}}/y^{\text{m}}_{\text{CO2}} - y^{\text{i}}_{\text{CH4}}/y^{\text{m}}_{\text{CH4}}}{N^{\text{ads}}_{\text{CO2},\mathbf{B1}}\left(1/y^{\text{m}}_{\text{CO2}} - 1/y^{\text{m}}_{\text{CH4}} \dfrac{y^{\text{i}}_{\text{CH4}}/y^{\text{i}}_{\text{CO2}}}{S^{\text{ads}}_{\mathbf{B1}}}\right)} F^{\text{i}}_{\text{mix},\mathbf{B}}. \quad \text{(S21)}$$

The molar flow rate of the product CH_4 can be obtained as:

$$F^{\text{o}}_{\text{CH4},\mathbf{B}} = y^{\text{i}}_{\text{CH4}} F^{\text{i}}_{\text{mix},\mathbf{B}} - \frac{L_{\mathbf{B2}} A_{\mathbf{B2}}(1 - \varepsilon_{\mathbf{B2}})}{\theta_{\mathbf{B}}} N^{\text{ads}}_{\text{CO2},\mathbf{B2}} \left\{\frac{y^{\text{m}}_{\text{CH4}}/y^{\text{m}}_{\text{CO2}}}{S^{\text{ads}}_{\mathbf{B2}}} + \alpha_{\mathbf{B2}}(1 - R_{\mathbf{B2}})\right\}$$
$$- \frac{L_{\mathbf{B1}} A_{\mathbf{B1}}(1 - \varepsilon_{\mathbf{B1}})}{\theta_{\mathbf{B}}} N^{\text{ads}}_{\text{CO2},\mathbf{B1}} \left\{\frac{y^{\text{i}}_{\text{CH4}}/y^{\text{i}}_{\text{CO2}}}{S^{\text{ads}}_{\mathbf{B1}}} + \alpha_{\mathbf{B1}}(1 - R_{\mathbf{B1}})\right\}, \quad \text{(S22)}$$

where, the adsorption properties of Column **B2**, $N^{\text{ads}}_{\text{CO2},\mathbf{B2}}$, $S^{\text{ads}}_{\mathbf{B2}}$, and $R_{\mathbf{B2}}$, are saturated values for the feed gas.

Supplementary Note 2: Comparison of System A and System B

Here, we focus on the adiabatic separation process of CO_2 described in the text, and assume that columns **A** and **B2** are filled with HKUST-1 and that column **B1** is filled with ELM-11. The adsorption characteristics of HKUST-1 should depend on the composition of a gas mixture. Therefore we investigated effective adsorption properties of column **B2** for the slipping-off gas as shown in Supplementary Figure 15, and obtained $N_{\mathrm{CO2},\mathbf{B2}}^{\mathrm{ads}}$ = 0.53 mmol/cm^3, $S_{\mathbf{B2}}^{\mathrm{ads}}$ = 5.35, and $R_{\mathbf{B2}}$ = −1.19 by using the composition of the intermediate flow between columns **B1** and **B2** ($y_{\mathrm{CO2}}^{\mathrm{m}} : y_{\mathrm{CH4}}^{\mathrm{m}} = 4.2 : 95.8$ at 263 K), which is obtained from $y_{\mathrm{CO2}}^{\mathrm{m}} = P^{\mathrm{gate}}/P_{\mathrm{t}}$. The negative value of $R_{\mathbf{B2}}$ comes from the fact that the amount of adsorbed CO_2 on HKUST-1 after the adiabatic adsorption process is smaller than that after the adiabatic desorption process. This is because only a small amount of slipped-off CO_2 is adsorbed on HKUST-1 after the adsorption process; however, more CO_2 is adsorbed at the rinsing process with pure CO_2 of 250 kPa.

For a fair comparison between systems **A** and **B**, we assumed the same void fraction, cross-sectional areas, and operating time for all the columns ($\varepsilon_{\mathbf{A}} = \varepsilon_{\mathbf{B1}} = \varepsilon_{\mathbf{B1}}$, $A_{\mathbf{A}} = A_{\mathbf{B1}} = A_{\mathbf{B2}}$, and $\theta_{\mathbf{A}} = \theta_{\mathbf{B}}$). Then, the proportionality factors for purging, $\alpha_{\mathbf{A}}$, $\alpha_{\mathbf{B1}}$, and $\alpha_{\mathbf{B2}}$, were set to be 1.0, which means that the amount of CH_4 required to purge the system is the same with the adsorbed amount of CO_2. We obtained $L_{\mathbf{B1}} = 0.325L_{\mathbf{A}}$, $L_{\mathbf{B2}} = 0.097L_{\mathbf{A}}$, and $L_{\mathbf{B2}} / L_{\mathbf{B1}} = 0.299$ in Case I by substituting the separation characteristics and mole fraction in each stream into Eqns. S9, S13 and S18, and $L_{\mathbf{B1}} = 0.224L_{\mathbf{A}}$, $L_{\mathbf{B2}} = 0.082L_{\mathbf{A}}$, and $L_{\mathbf{B2}} / L_{\mathbf{B1}}$ = 0364. in Case II from Eqns. S9, S21 and S24. The resulting material balances for systems **A** and **B** are depicted in Supplementary Figure 16.

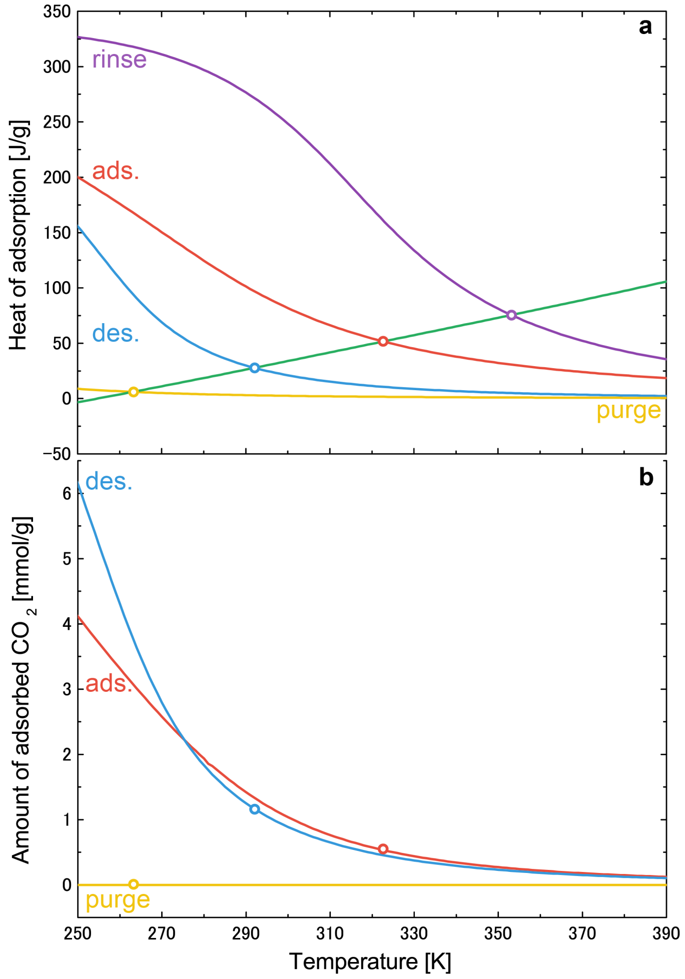

Supplementary Figure 15: Heat of adsorption and adsorption loadings in column B2 in case II. a Temperature dependence of the heat of adsorption for HKUST-1 on the four operating processes. The green line is the heat balance curves when the adiabatic adsorption process starts from 263 K. **b** Temperature dependence of the amount of adsorbed CO_2 on HKUST-1 for CO_2/CH_4 mixture (4.2:95.8) of 500 kPa (red), pure CO_2 of 15 kPa (blue), and pure CH_4 of 15 kPa (orange).

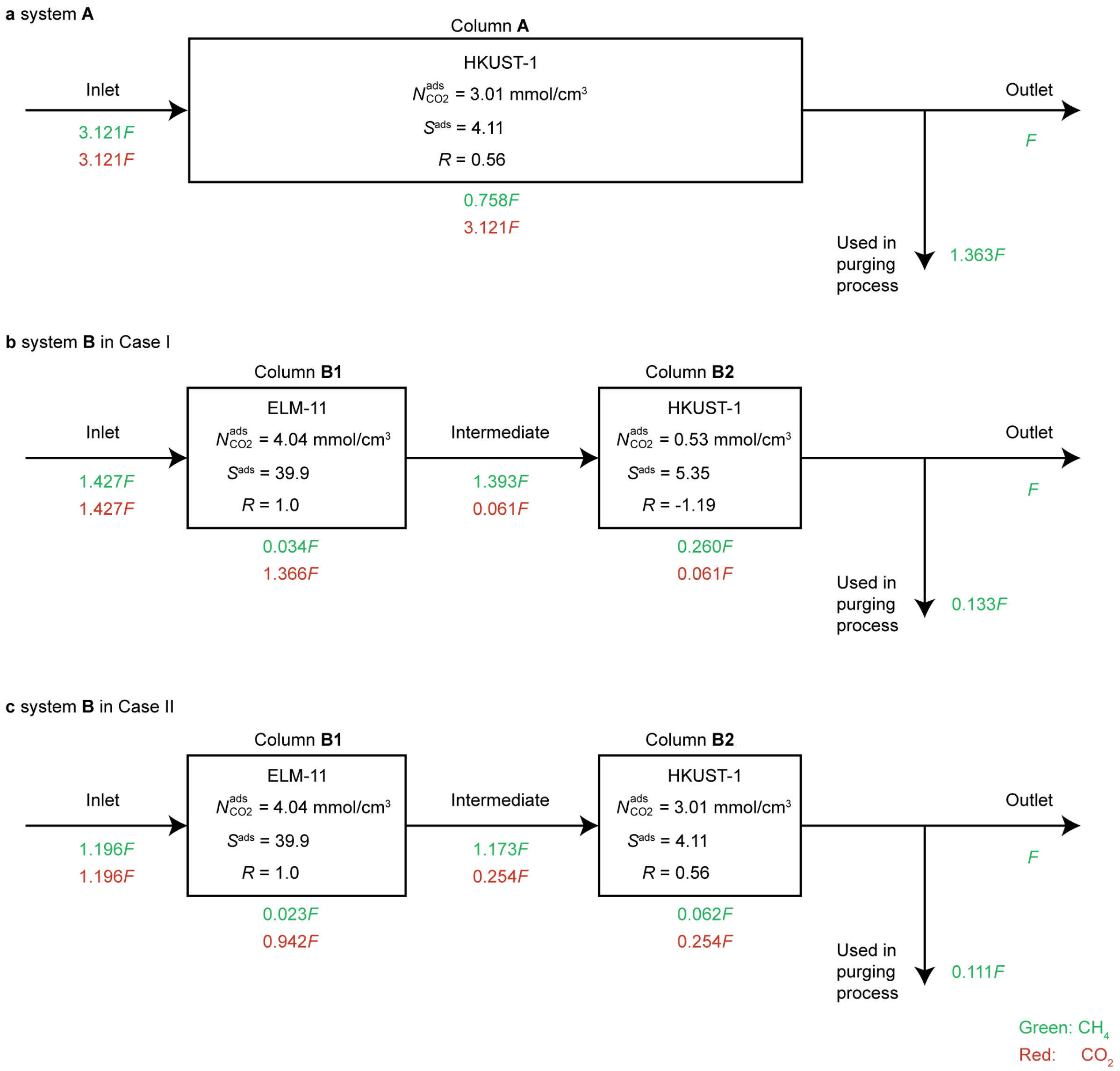

Supplementary Figure 16: The material balance and separation characteristics for system A and system B. The molar flow in red and green are for CO_2 and CH_4, respectively.
