# Supplementary material for: High-throughput gas separation by flexible metal–organic frameworks with fast gating and thermal management capabilities
Source: Nat Commun. 2020 Aug 3;11:3867. doi: 10.1038/s41467-020-17625-3 (PMC7400644; doi:10.1038/s41467-020-17625-3)
Supplement: Supplementary file 2 — Peer Review File [file 41467_2020_17625_MOESM2_ESM.docx]

Peer Review Information

**Journal:** Nature Communications

**Manuscript title:** High-throughput gas separation by flexible metal–organic frameworks with fast gating and thermal management capabilities

**Corresponding author name(s):** Prof Hideki Tanaka

**Editorial notes:**

**none**

**Reviewer comments & decisions:**

**Reviewer comments, first version:**

Reviewer #1 (Remarks to the Author):

The manuscript is on the very interesting concept of using flexible nanoporous materials for gas separation. It focuses specifically on including the thermal component of modeling of the adsorption and desorption processes in the material, something which is novel and has not really be done before. This is thus an interesting and novel study. There are, however, many points in the paper where the conclusions reached are not fully backed by facts, and where the modeling is insufficiently justified and/or has not been fully tested before it is applied. In this current state, the manuscript is not publishable.

Three major points that need further work to demonstrate the validity of the approach:

* The agreement between modeling and experiments in ELM-11 is rather poor. Take for example CH4, Figure 1C, the bad agreement is hidden by the scale of the graph. The authors should figure out the source of the agreement, and comment on it (instead of saying the agreement is good). They should further refine the model to obtain better agreement, too.

* The main difference between modeling and experiments is that the authors' GCMC modeling gives rise to infinitely steep steps upon structural transition. It is important that the authors study and detail the impact of these infinitely steep transitions, which are unphysical. They probably influence a lot the column modeling, whose partial differential equations are very sensitive to large-scale changes like that.

* The authors combine GCMC simulations for coadsorption, but it is not clear how they determine the transition point from this. I suppose they are using a free energy method similar to what they have used in previous work, but it is not clear. Is the transition occurring at thermodynamic equilibrium, or as the authors claimed in previous work, when the free energy barrier is sufficiently lowered compared to kT (thermal energy)? This is a very important point.


More minor points:

* The discussion section is extremely short (one paragraph) and lacks depth. It should describe both the limitations of the current methodology, as well as potential for further application.

* Also, the validity of GCMC simulations for the coadsorption transition needs to be checked against thermodynamic modeling.

* The references cited for previous work on structural transitions under adsorption do not show a complete picture of the literature on the topic. The ratio of self-citation is very high, with other authors' work on the topic being completely ignored (groups like Sholl, Fuchs, Snurr/Duren, etc.)

* The manuscript is written in an overly dramatic manner, with lots of useless meliorative adjectives or adverbs (very, excellent, ...). Even in the abstract, expressions like "an exquisitely combined column system" are out of place in a scientific paper.


Reviewer #2 (Remarks to the Author):

The manuscript from Hiraide et al. presents a process configuration for using flexible metal-organic framework (MOF) materials and avoid fast breakthrough of gas before the gate opening happens. The layered adsorbent configuration proposed is, as the authors claim "exquisite". That is the reason why is used industrially in many other applications. In hydrogen purification for example, three different layers of materials are used in the same columns. The objective there is to maximize the local adsorption of different gases. In gas dryers, two or three layers are also used. In this case, the fraction of water is successively reduced and materials with larger capacity at lower partial pressures are used. Alternatively, layered PSA units have been used to control the mass transfer zone inside PSA columns (same as here). This has been reported for air purification (1) and for biogas upgrading (2). The only novelty here is that is has been applied to gate-opening MOFs. Indeed, it will be important that the authors mention that layered PSA units are already applied industrially.

The authors should also explain better the definition of "theoretical limit" of a PSA. All studies indicate that the theoretical (and practical) limit is given by diffusion, but this topic has been not even mentioned here. Please refer to any of the publications from Prof. Sircar. (3 as an example). Also, if the PSA is taken to the limit, the flow profiles will not be as linear as indicated in Figure 4. If there are no diffusional limitations (as implicitly assumed by the authors here), the concentration and the temperature waves inside the column will travel together.

I am also not certain that the calculations of the HKUST-1 material in terms of thermal management are correct. They are only made for CO2 adsorption, but in this material, the CH4 adsorption is also important. What will happen to the temperature increase of HKUST-1 in the layered configuration? This should also be discussed.

Another important comment is regarding the first sentence of the abstract and what is done in the manuscript. The manuscript starts saying: "The enormous cost associated with CO2 separation…" Industrially, regulating the temperature of the stream to be separated to the ideal temperature where the material studied is suitable is one cause of enormous cost. In this case (as stated in page 6, line 216), the temperature is 263K (below freezing point of water) so other media should be used to cool the gas. A comment on this subject is expected.

The authors should correct the "regenerability" concept. Another name should be found. Regenerability sounds like the material cannot be regenerated, while this is not the case. Here, HKUST-1 is only partially loaded at the regeneration conditions.

Finally, and more importantly, the authors mention over 10 references regarding ELM material. I could not see any experimental data in this manuscript regarding breakthrough curves. I would indeed like to see the data that supports table 3. As written in page 5, line 179, "three data sets" mean that there is data generated and I would like to assume is experimental.
The reason why I'm mentioning a very practical (eventually considered imaginative limitation) is that for this particular case of flexible structures, there are several unknowns that might indeed put other process limits to the ones considered here; all methods for shaping used until today are for rigid materials. What happens if we use a rigid structure for supporting a flexible MOF? Do they break, or do they lose their gate effect? Also, what happens to the process if somehow, we manage to make flexible structures? Is the column going to be partially empty from the very beginning? Is the porosity going to change and create a pressure drop that will make the process impossible to handle?

Based on all these observations, I'm not sure that the manuscript is suitable for being published in Nature Communications. In case it is considered for publication, it will be necessary to make major modifications to grant that the right concepts and novelty are clearly stated.


References:
1. Lu, Y.; Doong, S-J.; Bülow, M. Pressure-Swing Adsorption Using Layered Adsorbent Beds with Different Adsorption Properties: II—Experimental Investigation. Adsorption, 2005, 10, 267
2. Grande, C. A.; Rodrigues, A. E. Layered Vacuum Pressure-Swing Adsorption for Biogas Upgrading. Ind. Eng. Chem. Res., 2007, 46, 7844.
3. Chai, S. W.; Kothare, M. V.; Sircar, S. Rapid Pressure Swing Adsorption for Reduction of Bed Size Factor of a Medical Oxygen Concentrator. Ind. Eng. Chem. Res., 2011, 50, 8703.


Reviewer #3 (Remarks to the Author):

The authors have attempted to design a rapid VSA system for CO2 separation from landfill source by using a combination of flexible and rigid MOFs in series. They claim that intrinsic thermal management capabilities of their choice of MOF (ELM-11) make it possible to use this material in a rapid VSA process without losing a lot of working capacity. They also use HKUST-1 in series with ELM-11 to capture the CO2 that does not get captured by the latter due to the ‘slipping off’ issue. Detailed calculations and experimental procedures for isotherm measurements, breakthrough measurements, XRD measurements, GCMC simulations, thermodynamic modeling, and system design for combining both MOFs are provided.
The reviewer has some concerns about the effectiveness of the process described by the authors. A lot of the description provided by the authors revolve around idealized calculations without verification with proper experimental backup. While intrinsic thermal management is theoretically promising; but in an actual system, the timescales for endothermic gate-opening and exothermic adsorption process along the direction of the feed flow would be significant. The authors assume that heat transfer between adsorbent crystals and that between the feed gas and adsorption column can be simplified or neglected, but the reviewer does not agree with this assumption. Without proper assessment of the timescales of heat transfer, it would be difficult to evaluate if this system can be used for ‘rapid’ VSA as the authors claim. In short, the authors have done a commendable job in modeling the thermodynamics of this process but not enough attention has been paid to the transient nature of these processes.
Some findings in this work are also shown without providing adequate explanations. For example, in page 3, line 117, it is written “Clearly, the rate of phase transition is increased with decreasing temperature under the same CO2 pressure of 40.9 ± 0.1 kPa (see Table 2).” There is no explanation given why this phase transition becomes faster with decreasing temperature.
Also in page 4, line 120 it is said “The rate of structural transition increased with increasing CO2 pressure, and surprisingly, the phase transition was completed within a few seconds at highest gas pressure.” There is no context provided here for the readers to understand why this finding is “surprising”.
Overall there is a lot of discussion in this paper that has been sequestered into the supporting information due to issues with length. It feels that this paper would be better suited as a full manuscript rather than in a communication format. Most of the discussion revolves around idealized calculations that are not backed up with proper experiments; for example – the combined ELM-11/HKUST-1 scheme is illustrated using a representative breakthrough curve without practical experimental considerations. While this is acceptable for a first pass analysis of a proposed system for landfill gas separation, it is the opinion of this reviewer that the manuscript in its present state is not fit for nature communications.

**Author rebuttal, first version:**

Reviewer #1:

The manuscript is on the very interesting concept of using flexible nanoporous materials for gas separation. It focuses specifically on including the thermal component of modeling of the adsorption and desorption processes in the material, something which is novel and has not really be done before. This is thus an interesting and novel study.

We thank reviewer #1 for the positive remarks regarding the novelty of our work.

There are, however, many points in the paper where the conclusions reached are not fully backed by facts, and where the modeling is insufficiently justified and/or has not been fully tested before it is applied. In this current state, the manuscript is not publishable.

Three major points that need further work to demonstrate the validity of the approach:

* The agreement between modeling and experiments in ELM-11 is rather poor. Take for example CH4, Figure 1C, the bad agreement is hidden by the scale of the graph. The authors should figure out the source of the agreement, and comment on it (instead of saying the agreement is good). They should further refine the model to obtain better agreement, too.

The theoretical (GCMC) and experimental isotherms of CH4 must be different because the GCMC isotherm corresponds to CH4 adsorption on the “open” framework of ELM-11, while the experimental isotherm corresponds to the one on the “closed” framework of ELM-11. The fictitious GCMC isotherm for CH4 on the quenched open framework shows less adsorption even though the framework has voids to accommodate CH4 molecules, which occurs due to the weak solid–fluid interactions. The experimental isotherm of CH4 shows no adsorption because the affinity of CH4 is insufficient to pry open the framework of ELM-11. To clarify this, we added the following sentence in the manuscript:

*The fictitious GCMC isotherm for CH4 on the quenched open framework shows low adsorption, even though the framework has voids to accommodate CH4 molecules, which is due to the weak solid–fluid interactions. In contrast, the experimental isotherm of CH4 shows no adsorption, which is because the affinity of CH4 is too weak to pry open the framework of ELM-11*

* The main difference between modeling and experiments is that the authors' GCMC modeling gives rise to infinitely steep steps upon structural transition. It is important that the authors study and detail the impact of these infinitely steep transitions, which are unphysical. They probably influence a lot the column modeling, whose partial differential equations are very sensitive to large-scale changes like that.

The column modelling used in this study is not based on partial differential equations, and thus it does not affect our results, which should assuage the reviewer’s concern. Additionally, the infinitely steep transition is not unphysical because the gate adsorption is a first-order phase transition.

* The authors combine GCMC simulations for coadsorption, but it is not clear how they determine the transition point from this. I suppose they are using a free energy method similar to what they have used in previous work, but it is not clear. Is the transition occurring at thermodynamic equilibrium, or as the authors claimed in previous work, when the free energy barrier is sufficiently lowered compared to kT (thermal energy)? This is a very important point.

The gate adsorption/desorption pressures on the coadsorption of CO2 and CH4 were predicted based on the experimental gate-opening/closing pressures for pure CO2 as described in Supplementary Data 3, and not by using the theoretical calculations based on the free energy method. The validity of the assumption that the gate adsorption/desorption pressures can be evaluated from the composition of CO2 and CH4 in the gas mixture was supported and confirmed using XRPD and breakthrough curve measurements, as mentioned in the manuscript

.

More minor points:

* The discussion section is extremely short (one paragraph) and lacks depth. It should describe both the limitations of the current methodology, as well as potential for further application.

We enriched the discussion section accordingly.

* Also, the validity of GCMC simulations for the coadsorption transition needs to be checked against

thermodynamic modeling.

As mentioned above, the coadsorption transitions were predicted using experimental results. The thermodynamic method based on the GCMC simulations was not used.

* The references cited for previous work on structural transitions under adsorption do not show a complete picture of the literature on the topic. The ratio of self-citation is very high, with other authors' work on the topic being completely ignored (groups like Sholl, Fuchs, Snurr/Duren, etc.)

We have cited papers from the groups suggested by reviewer #1, and accordingly removed the references regarding

ELM-11.

* The manuscript is written in an overly dramatic manner, with lots of useless meliorative adjectives or adverbs (very, excellent, ...). Even in the abstract, expressions like "an exquisitely combined column system" are out of place in a scientific paper.

We have addressed such expressions in the manuscript accordingly.

Reviewer #2:

The manuscript from Hiraide et al. presents a process configuration for using flexible metal-organic framework (MOF) materials and avoid fast breakthrough of gas before the gate opening happens. The layered adsorbent configuration proposed is, as the authors claim "exquisite". That is the reason why is used industrially in many other applications. In hydrogen purification for example, three different layers of materials are used in the same columns.The objective there is to maximize the local adsorption of different gases. In gas dryers, two or three layers are also used. In this case, the fraction of water is successively reduced and materials with larger capacity at lower partial pressures are used. Alternatively, layered PSA units have been used to control the mass transfer zone inside PSA columns (same as here). This has been reported for air purification (1) and for biogas upgrading (2). The only novelty here is that is has been applied to gate-opening MOFs. Indeed, it will be important that the authors mention that layered PSA units are already applied industrially.

We thank reviewer #2 for the comment. We added the following statement in the manuscript:

*To prevent the CO2 slipping-off problem, we propose attaching a secondary column containing a conventional*

*adsorbent that exhibits a Langmuir-type CO2 isotherm to a primary column filled with the flexible MOF. Although this system is similar to the one with multi-layered columns for separating a mixture of three or more components,35 it is based on a new strategy using multiple items to improve the efficiency of the rapid PVSA system using the flexible MOF, as described in the following sections.*

The concept of our proposed system is different from that of a multi-layered column system typically used in the industry, although their structures are similar. The secondary column filled with conventional porous materials (including rigid MOFs) can be designed not only to process the slipping-off CO2 but also to enable the maximal utilization of the fast gating of flexible MOFs. Specifically, the primary column filled with flexible MOFs can only be used to treat a gas mixture with a high concentration of CO2 providing a large *P*−*P*gate, which results in fast gating. Moreover, the efficiency of the sequential-column system can be further improved if the operations for the primary

and secondary columns are optimized and performed individually. For example, the desorption process for the primary column and the purging process for the secondary column can be performed simultaneously, which reduces the total cycle time.

The authors should also explain better the definition of "theoretical limit" of a PSA. All studies indicate that the theoretical (and practical) limit is given by diffusion, but this topic has been not even mentioned here.

We removed the words “theoretical limit” and revised the manuscript for a more specific description.

Please refer to any of the publications from Prof. Sircar. (3 as an example).

We cited the papers by Prof. Shivaji Sircar’s group including reference 3 suggested by the reviewer.

Also, if the PSA is taken to the limit, the flow profiles will not be as linear as indicated in Figure 4. If there are no diffusional limitations (as implicitly assumed by the authors here), the concentration and the temperature waves inside the column will travel together.

The reviewer’s comment is slightly unclear; however, Figure 4 (Figure 5 in this revised paper) shows the heat of adsorption, not the flow profiles. We agree with the reviewer’s indication that the concentration and the temperature waves inside the column travel together; however, our calculations/results do not contradict it.

I am also not certain that the calculations of the HKUST-1 material in terms of thermal management are correct. They are only made for CO2 adsorption, but in this material, the CH4 adsorption is also important.

We are afraid that there is a misunderstanding. The heat release and absorption on HKUST-1 were thoroughly evaluated for coadsorption of CO2 and CH4 without ignoring CH4 adsorption. The details are provided in Supplementary Data 1.

What will happen to the temperature increase of HKUST-1 in the layered configuration? This should also be discussed.

The temperature of HKUST-1 in the secondary column increases when it adsorbs the slipping-off CO2 as well as CH4, and this effect was strictly incorporated in our calculations. Details are provided in Supplementary Method 1.

Another important comment is regarding the first sentence of the abstract and what is done in the manuscript. The manuscript starts saying: "The enormous cost associated with CO2 separation…" Industrially, regulating the temperature of the stream to be separated to the ideal temperature where the material studied is suitable is one cause of enormous cost. In this case (as stated in page 6, line 216), the temperature is 263K (below freezing point of water) so other media should be used to cool the gas. A comment on this subject is expected.

The comment by the reviewer is reasonable as we noted a lack of explanation in the previous manuscript. Assuming that the system is completely adiabatic, the cost of cooling is unnecessary once the system was cooled down to the starting temperature and the cyclic steady state was established; hence, for a partially heat-insulated PVSA system, a very low amount of energy for cooling would be required to maintain the cyclic steady state. We added the following sentences in the manuscript:

*It should be also noted that, assuming that the system is completely adiabatic, the additional cost of cooling is*

*unnecessary once the system cools down to the starting temperature and the cyclic steady state is established; hence, for a partially heat-insulated PVSA system, only a small amount of energy would be required for cooling to maintain the cyclic steady state*

*.*

The authors should correct the "regenerability" concept. Another name should be found. Regenerability sounds like the material cannot be regenerated, while this is not the case. Here, HKUST-1 is only partially loaded at the regeneration conditions.

The term “regenerability” can be found in papers concerning PSA and it has been used with the same connotation as in our manuscript; e.g., reference 13 by Bae *et al.*; Prats, H., Bahamon, D., Giménez, X., Gamallo, P. & Sayós, R., Computational simulation study of the influence of faujasite Si/Al ratio on CO2 capture by temperature swing adsorption. *J. CO2 Util*. **21**, 261–269, (2017); Yang, Q., Liu, D., Zhong, C. & Li, J. Development of Computational Methodologies for Metal–Organic Frameworks and Their Application in Gas Separations. *Chem. Rev.* **113**, 8261– 8323, (2013); Tagliabue, M. et al. Regenerability of zeolites as adsorbents for natural gas sweetening: A case-study. *Fuel* **93**, 238–244 (2012).

Finally, and more importantly, the authors mention over 10 references regarding ELM material.

I could not see any experimental data in this manuscript regarding breakthrough curves.

As mentioned in the reply to reviewer #1, we reduced the citations concerning ELM-11 accordingly and added the experimental breakthrough curves for the sequential-column system consisting of ELM-11 and HKUST-1.

I would indeed like to see the data that supports table 3. As written in page 5, line 179, "three data sets" mean that there is data generated and I would like to assume is experimental.

The three data sets were estimated using the experimental results based on the simple and basic thermodynamics. As for HKUST-1, the adsorption isotherms, the heat of adsorption, and the specific heat, which are required for the estimation, were obtained from literature. In case of ELM-11, data were obtained using GCMC simulations and DFT calculations based on the structure model of ELM-11 determined in our previous works. The validity of simulating the adsorption characteristics using the structure model was established with references 9, 19, and 22. To obtain the three data sets directly by experiments, the construction of a PSA system is required, which is beyond the scope of this study, to show the possibility that the intrinsic thermal management and the pressure-aided fast

gating capabilities of flexible MOFs are useful for increasing the efficiency of the rapid PVSA.

The reason why I'm mentioning a very practical (eventually considered imaginative limitation) is that for this particular case of flexible structures, there are several unknowns that might indeed put other process limits to the ones considered here; all methods for shaping used until today are for rigid materials. What happens if we use a rigid structure for supporting a flexible MOF? Do they break, or do they lose their gate effect? Also, what happens to the process if somehow, we manage to make flexible structures? Is the column going to be partially empty from the very beginning? Is the porosity going to change and create a pressure drop that will make the process impossible to handle?

We thank reviewer #2 for all the questions and have tried to resolve them to realize the rapid PVSA using flexible MOFs. We added the following sentences in the Discussion section:

*It should be also noted that, to realize rapid PVSA using the flexible MOFs, some issues must be resolved, for*

*example, the hindrance of gate-opening by the pelletization of flexible MOFs, pellets braking owing to the gateopening,*

*and a pressure drop owing to the volume expansion of the pellets induced by the gate-opening. We have*

*already started addressing these concerns, and the results will be published elsewhere.*

References:

1. Lu, Y.; Doong, S-J.; Bülow, M. Pressure-Swing Adsorption Using Layered Adsorbent Beds with Different Adsorption Properties: II—Experimental Investigation. Adsorption, 2005, 10, 267

2. Grande, C. A.; Rodrigues, A. E. Layered Vacuum Pressure-Swing Adsorption for Biogas Upgrading. Ind. Eng.Chem. Res., 2007, 46, 7844.

3. Chai, S. W.; Kothare, M. V.; Sircar, S. Rapid Pressure Swing Adsorption for Reduction of Bed Size Factor of a Medical Oxygen Concentrator. Ind. Eng. Chem. Res., 2011, 50, 8703.

Reviewer #3:

The authors have attempted to design a rapid VSA system for CO2 separation from landfill source by using a combination of flexible and rigid MOFs in series. They claim that intrinsic thermal management capabilities of their choice of MOF (ELM-11) make it possible to use this material in a rapid VSA process without losing a lot of working capacity. They also use HKUST-1 in series with ELM-11 to capture the CO2 that does not get captured by the latter due to the ‘slipping off’ issue. Detailed calculations and experimental procedures for isotherm measurements, breakthrough measurements, XRD measurements, GCMC simulations, thermodynamic modeling, and system design for combining both MOFs are provided. The reviewer has some concerns about the effectiveness of the process described by the authors. A lot of the description provided by the authors revolve around idealized calculations without verification with proper experimental backup. While intrinsic thermal management is theoretically promising; but in an actual system, the timescales for endothermic gate-opening and exothermic adsorption process along the direction of the feed flow would be significant. The authors assume that heat transfer between adsorbent crystals and that between the feed gas and adsorption column can be simplified or neglected, but the reviewer does not agree with this assumption. Without proper assessment of the timescales of heat transfer, it would be difficult to evaluate if this system can be used for ‘rapid’ VSA as the authors claim. In short, the authors have done a commendable job in modeling the thermodynamics of this process but not enough attention has been paid to the transient nature of these processes.

We thank reviewer #3 for rating our job as commendable. As mentioned above, we added the breakthrough experiments to show the feasibility of the sequential-columns system by using flexible MOFs. The timescales of heat transfer for the endothermic gate-opening and exothermic adsorption should be exactly the same because the generation of endothermic and exothermic heat occurs inside the same crystal particle of ELM-11. Heat transfer between the host framework and the adsorbed guest molecules can be immediately performed because of the frequent collisions between them; therefore, the intrinsic thermal management of flexible MOFs is promising. Thus,

we believe that neglecting the heat transfer between the adsorbent crystals and between the feed gas and the adsorption column should provide a good approximation because the corresponding timescales of heat transfer are relatively slow compared with the cycle time assumed in the rapid PVSA.

Some findings in this work are also shown without providing adequate explanations. For example, in page 3, line 117, it is written “Clearly, the rate of phase transition is increased with decreasing temperature under the same CO2 pressure of 40.9 ± 0.1 kPa (see Table 2).” There is no explanation given why this phase transition becomes faster with decreasing temperature. Also in page 4, line 120 it is said “The rate of structural transition increased with increasing CO2 pressure, and surprisingly, the phase transition was completed within a few seconds at highest gas pressure.” There is no context provided here for the readers to understand why this finding is “surprising”.

We revised the entire manuscript. Clarifying how the rate of phase transition increased with decreasing temperature is not within the scope of this work; however, we believe that this is because the *P*−*P*gate value, which can be regarded as a driving force for the phase transition, increases with decreasing temperature at a constant gas pressure *P* (the *P*gate value decreases with decreasing temperature).

Overall there is a lot of discussion in this paper that has been sequestered into the supporting information due to issues with length. It feels that this paper would be better suited as a full manuscript rather than in a communication format.

We believe that the communication format is appropriate to demonstrate the novelty of our concept and that our supplementary information is necessary for readers interested in following our study.

Most of the discussion revolves around idealized calculations that are not backed up with proper experiments; for example – the combined ELM-11/HKUST-1 scheme is illustrated using a representative breakthrough curve without practical experimental considerations. While this is acceptable for a first pass analysis of a proposed system for landfill gas separation, it is the opinion of this reviewer that the manuscript in its present state is not fit for nature communications.

As mentioned above, we added the results from breakthrough measurements for the sequential-column system using ELM-11 and HKUST-1 and demonstrated that our strategy is feasible.

**Reviewer comments, second version:**

Reviewer #1 (Remarks to the Author):

This important revision has vastly improved the manuscript, and the addition of breakthrough experiments significantly expand the impact of the work. My points have been addressed and I support publication.


Reviewer #2 (Remarks to the Author):

The authors have modified the manuscript considerably and have addressed most of the issues raised. I think that the manuscript is of a very high quality and can be published. Only minor comments from my side. I guess that two things that may be useful for the manuscript are missing:
1- temperature profiles in the breakthrough curves. This can be shown in the Supplementary information but it can allow the reader to evaluate how adiabatic the system is and to see the thermal evolution of the system. In the submitted manuscript you have shown the mass transfer, but as your concept shows, mass and heat transfer are strongly interlinked.
2- If the authors can mention the heat capacity of the MOF materials it can be very useful.
I want to congratulate the authors for the results achieved and for the nice presentation of the idea of using a layered PSA to overcome the gate-opening "slipping" phenomenon.
With my best regards,
Carlos A. Grande


Reviewer #3 (Remarks to the Author):

The authors have done a commendable job on revising their manuscript based on the feedback from multiple reviewers. They have provided adequate clarifications wherever necessary and revised the manuscript accordingly. This reviewer has no major concerns about the scientific and technical aspects of this manuscript currently. However, the feeling still remains that the discussion section is still very condensed and glossed over. This is likely a side effect of this manuscript being a Communications manuscript and not a full length article. I would encourage the authors and editor to reconsider the decision to proceed with the Communications format.

**Author rebuttal, second version:**

Reviewer #1 (Remarks to the Author):

This important revision has vastly improved the manuscript, and the addition of breakthrough experiments significantly expand the impact of the work. My points have been addressed and I support publication.

Author reply:

We thank the reviewer #1 for validating the importance of our work and approving the revised manuscript.

Reviewer #2 (Remarks to the Author):

The authors have modified the manuscript considerably and have addressed most of the issues raised. I think that the manuscript is of a very high quality and can be published.

Author reply:

We thank the reviewer #2 for validating the importance of our work and for approving the revised manuscript.

Only minor comments from my side. I guess that two things that may be useful for the manuscript are missing:

1- temperature profiles in the breakthrough curves. This can be shown in the Supplementary information but it can allow the reader to evaluate how adiabatic the system is and to see the thermal evolution of the system. In the submitted manuscript you have shown the mass transfer, but as your concept shows, mass and heat transfer are strongly interlinked.

Author reply:

As the reviewer pointed out, the mass and heat transfers are strongly interlinked. Therefore, we performed the breakthrough curve measurements under "isothermal" condition to clearly and accurately show that the slipping-off can be well predicted by the gate-opening pressure and the slipping-off problem can be solved by using the sequential-column system. We are currently developing a column to precisely measure the thermal evolution of the system under adiabatic conditions and the experimental results will be reported soon.

2- If the authors can mention the heat capacity of the MOF materials it can be very useful.

Author reply:

The specific heat capacities of ELM-11 and HKUST-1 as a function of temperature have been provided in the

Supplementary Information section. We also added the following sentences in the Method section:

*The obtained phonon density of states and specific heat of ELM-11 as a function of temperature are shown in Supplementary Figures 8 and 9, respectively. The regular specific heat of ELM-11 in the open state at 298 K,*

*obtained using this calculation, was 0.94 J g^−1^ K^−1^.*

*We fitted a polynomial function to the experimental specific heats of HKUST-1 reported by Kloutse et al.^29^ (below 320 K) and Mu et al.^30^ (over 333 K), and the function was integrated with respect to temperature to evaluate the heat required to change the temperature of the host framework (green plane in Figure 5a). The parameters of the polynomial function are listed in Supplementary Table 6, and the specific heat of HKUST-1 as a function of temperature is shown in Supplementary Figure 9. The regular specific heat of HKUST-1 at 298 K was 0.78 J g−1 K^−1^.*

I want to congratulate the authors for the results achieved and for the nice presentation of the idea of using a layered

PSA to overcome the gate-opening "slipping" phenomenon.

With my best regards,

Carlos A. Grande

Author reply:

We thank Prof. Grande for his favorable assessment of our research and for all his fruitful comments.

Reviewer #3 (Remarks to the Author):

The authors have done a commendable job on revising their manuscript based on the feedback from multiple reviewers. They have provided adequate clarifications wherever necessary and revised the manuscript accordingly. This reviewer has no major concerns about the scientific and technical aspects of this manuscript currently. However, the feeling still remains that the discussion section is still very condensed and glossed over. This is likely a side effect of this manuscript being a Communications manuscript and not a full length article. I would encourage the authors and editor to reconsider the decision to proceed with the Communications format.

Author reply:

We thank the reviewer #3 for the positive remarks and the comment about the "Discussion" section. We carefully reread the manuscript and moved a portion of the "Strategy for preventing the slipping-off problem" section to the "Discussion" section and also added some important explanations to enhance clarity. The sentences added in the "Discussion" section are as follows:

*It is worth noting that our proposed sequential-column system is suitable for flexible MOFs showing pressure-aided fast gating because the primary column containing a flexible MOF can be designed to treat a gas mixture with a high concentration of CO2 or a large P − Pgate value. The secondary column should therefore be designed to process the slipping-off CO2 and to maximize the utilization of fast gating of the flexible MOFs. Further, note that filling a simple mixture of a flexible MOF and a rigid nanoporous material into one adsorption column cannot inherently solve the slipping-off problem. Mixing the materials only produces a type IV isotherm (IUPAC classification^38^) as a sum of the gate adsorption and Langmuir-type isotherms, which cannot avoid the two-stage breakthrough of CO2, although a modest amount of pure CH4 can be obtained before the first breakthrough of CO2 (see Supplementary Figure 5 for more details).*

**Reviewer comments, third version:**

**Author rebuttal, third version:**
